# Establishing a prognostic model based on immune-related genes and identification of BIRC5 as a potential biomarker for lung adenocarcinoma patients

**DOI:** 10.1186/s12885-023-11249-8

**Published:** 2023-09-23

**Authors:** Qianhe Ren, Qifan Li, Chenye Shao, Pengpeng Zhang, Zhuangzhuang Hu, Jun Li, Wei Wang, Yue Yu

**Affiliations:** 1 Department of Thoracic Surgery, The First Affiliated Hospital of Nanjing Medical University, Nanjing, China; 2 Department of Thoracic Surgery, The First Affiliated Hospital of Soochow University, Suzhou, China; 3 Department of Urology, Shuyang First People’s Hospital, Suqian, China

**Keywords:** lung adenocarcinoma, Immune genes, Risk signature, Prognosis, Tumor mutation burden, Immunotherapy

## Abstract

**Background:**

Lung adenocarcinoma (LUAD) is an extraordinarily malignant tumor, with rapidly increasing morbidity and poor prognosis. Immunotherapy has emerged as a hopeful therapeutic modality for lung adenocarcinoma. Furthermore, a prognostic model (based on immune genes) can fulfill the purpose of early diagnosis and accurate prognostic prediction.

**Methods:**

Immune-related mRNAs (IRmRNAs) were utilized to construct a prognostic model that sorted patients into high- and low-risk groups. Then, the prediction efficacy of our model was evaluated using a nomogram. The differences in overall survival (OS), the tumor mutation landscape, and the tumor microenvironment were further explored between different risk groups. In addition, the immune genes comprising the prognostic model were subjected to single-cell RNA sequencing to investigate the expression of these immune genes in different cells. Finally, the functions of BIRC5 were validated through in vitro experiments.

**Results:**

Patients in different risk groups exhibited sharply significant variations in OS, pathway activity, immune cell infiltration, mutation patterns, and immune response. Single-cell RNA sequencing revealed that the expression level of BIRC5 was significantly high in T cells. Cell experiments further revealed that BIRC5 knockdown markedly reduced LUAD cell proliferation.

**Conclusion:**

This model can function as an instrumental variable in the prognostic, molecular, and therapeutic prediction of LUAD, shedding new light on the optimal clinical practice guidelines for LUAD patients.

**Supplementary Information:**

The online version contains supplementary material available at 10.1186/s12885-023-11249-8.

## Introduction

As an extremely malignant tumor, lung cancer is among the most commonly diagnosed cancers (11.6% of the total cases) and is the leading cause of cancer death (18.4% of all cancer deaths) [1]. All lung cancers consist of two main subtypes: small-cell lung carcinoma (SCLC) and non-small cell lung carcinoma (NSCLC) [2]. Accounting for over 40% of non-small cell lung cancers, lung adenocarcinoma (LUAD) is overwhelmingly the most common histologic type of lung cancer [3]. The most advantageous approach for managing patients with locally advanced non-small cell lung cancer (NSCLC) that is amenable to surgical resection involves administering chemoradiation as a minimum [4]. The utilization of trimodality treatment, which encompasses surgical resection, has been a contentious topic for numerous decades. Furthermore, for patients with inoperable or unresectable locally advanced disease, the adoption of immunotherapy consolidation following chemoradiation has established a novel benchmark for care. Despite improvements in surgery in recent years, the prognosis of lung cancer remains unfavorable. Thus, more comprehensive therapies are urgently needed. The development of cancer genomics in recent decades has permitted the identification of several gene alterations as driver gene mutations for LUAD, including anaplastic lymphoma kinase (ALK), epidermal growth factor receptor (EGFR) and KRAS [5–7]. Several therapies that target these gene alterations have been employed. EGFR is found to be mutated in as much as 59.7% of NSCLC tumors in Asian patients and approximately 16.7% of those in Caucasian patients. Novel therapeutic agents known as tyrosine kinase inhibitors (TKIs) have been developed to specifically target these mutations, including erlotinib, gefitinib, and afatinib, which have demonstrated response rates of up to 75% [8]. The mechanism of targeted therapy focused on these mutation sites involves the use of drugs that specifically inhibit the activity of the altered protein. For example, EGFR inhibitors, such as erlotinib and gefitinib, block the activity of the EGFR protein, preventing the activation of downstream signaling pathways that promote cell growth and survival. Similarly, ALK inhibitors, such as crizotinib and ceritinib, block the activity of the ALK protein, which is often altered in lung cancer. In summary, the mechanism of targeted therapy focused on ALK, EGFR, KRAS, and other mutation sites involves the use of drugs that specifically inhibit the activity of the altered protein. These drugs can effectively target cancer cells while sparing healthy cells, reducing side effects, and improving patient outcomes. Despite improvements in the prognoses of some patients after receiving targeted treatments, a large number of patients eventually become resistant to targeted therapy [9]. For example, all patients possessing activating mutations in EGFR eventually encounter resistance to TKIs after a median duration of 12 months. The most prominent resistance mechanism observed is a secondary point mutation located in exon 20 of EGFR (T790M), wherein methionine is substituted by threonine at amino acid position 790 [10]. Under these circumstances, the advent of immunotherapy provides novel insight into lung cancer therapy.

The rapid development of cancer immunology in recent years has provided a novel perspective for cancer therapy [11]. A complex network has been well established to regulate interactions between the immune system and cancers. The human immune system can recognize and extinguish abnormal tumor cells. Immune checkpoint inhibitors (ICIs) have recently gained increasing attention as an essential part of immunotherapy [12]. Furthermore, tremendous advances in immune checkpoint blockade have introduced a paradigm shift in treatment for patients with lung cancer. In addition, immune checkpoint inhibitor (ICI) treatment has functioned as the standard of care for patients with extensive-stage small cell lung cancer or locally advanced/metastatic non-small cell lung cancer without EGFR/ALK alterations [13]. Thus, ICIs have been widely used in LUAD therapy. For example, immune checkpoint inhibitors (ICIs) that target programmed cell death 1 (PD-1) and programmed cell death-ligand 1 (PD-L1) play a significant role in the immune check-point pathway, exhibiting excellent and durable antitumor activity in LUAD patients [14]. The signaling pathway of programmed cell death 1 (PD-1) is often co-opted by malignant cells as a means of evading immunological scrutiny. Consequently, the PD-1 pathway serves to stifle T cell activities, such as their activation, proliferation, and production of cytokines. As it stands, antibodies that obstruct either PD-1 itself or its ligand, PD-L1, have gained regulatory approval for employment in treating an array of solid and hematologic neoplasms [15]. In addition to PD-1 and PD-L1, cytotoxic T-lymphocyte-associated antigen 4 (CTLA-4) also presents promising results in the treatment of advanced-stage lung cancer patients [16]. Despite the great impact of immunotherapy on the treatment of LUAD patients, many patients still experience disease progression during treatment or after treatment discontinuation due to immune resistance [17]. In immunocompetent individuals, neoplastic cells can undergo three outcomes: eradication, stasis, or evasion. Tumor immune evasion (TIE) refers to a mechanism by which the immune milieu of molded neoplasms can proliferate via an unbridled route [18]. The continual interactions amidst neoplastic cells and the neoplastic microenvironment are pivotal in neoplasm inception, advancement, metastasis, and reaction to therapeutic interventions [19]. The tumor microenvironment plays an important role in the immunotherapy response. Exploring the tumor microenvironment (TME) can improve the effect of cancer immunotherapy. Under these circumstances, constructing an immune gene signature is crucial to predict the prognosis and efficacy of LUAD immunotherapy.

In our study, a novel immune gene signature marker that can predict the response to immunotherapy was developed. After being validated by the GEO database, its prediction value in the prognosis of LUAD patients was proven to be excellent. Then, several clinicopathological characteristics were analyzed to explore the correlations between them and the prognostic model. To elucidate the TME of LUAD, the tumor mutation burden (TMB) and immune infiltration were further analyzed. In addition, the prediction of immunotherapy response and prognostic ability of various models were compared. Furthermore, BIRC5, an immune gene in the prognostic model, was identified to be significantly enriched in T cells by single-cell sequencing analysis. Finally, cell experiments were further performed to confirm the effects of BIRC5 on LUAD cells.

## Materials and methods

### Public data collection

Two public databases were leveraged in this study. RNA-seq data of 551 samples (497 tumor samples, 54 normal samples) with clinical characteristics and tumor mutation burden (TMB) were collected from The Cancer Genome Atlas (TCGA) database (https://portal.gdc.cancer.gov/),  functioningas the training set. Samples with an unknown total survival time were excluded. Two transcription profile datasets (GSE72094 and GSE26939), consisting of 512 samples in total, were obtained from GEO databases (https://www.ncbi.nlm.nih.gov/geo/) and used as validation sets. The criteria for messenger RNA (mRNA) expression data were set as log2 conversion, and the average expression amount was considered the gene expression quantity. Additionally, immune-related genes (IRGs) were obtained from IMMPORT. (https://www.immport.org/home) and InnateDB (https://www.innatedb.ca/).

### Differentially expressed immune-related genes

Differentially expressed genes between LUAD and corresponding normal tissues were analyzed based on TCGA data to screen out immune-related genes (IRGs) involved in oncogenesis. Aberrantly expressed genes were obtained using the ‘limma’ package [20–22] (|logFC |> 1 and false discovery rate (FDR) < 0.05). Then, differentially expressed IRGs were obtained by interacting IRGs and differentially expressed genes. Furthermore, the R package ‘ggplot2’ was utilized to complete the volcano map. The log2(fold change) was set to two to improve the reliability of the result in the volcano map.

### Weighted correlation network analysis (WGCNA)

Based on the principle of WGCNA calculation [23–25], highly coexpressed gene modules represent many specifically expressed genes that are significantly correlated with several tumors. To obtain the genes extraordinarily related to lung adenocarcinoma, weight correlation network analysis was further conducted. By using the R packages ‘WGCNA’ and ‘limma’, different modules containing coexpressed IRGs were obtained. The modules were named by different colors, and the number represents the significance of the difference between tumor samples and normal samples.

### Constructing an IRG‑related prognostic model for LUAD

First, the expression of the coexpressed genes in TCGA and GEO was obtained by intersecting the transcriptome profile collected from TCGA data and GEO data. Then, based on the weighted correlation network analysis, IRGs in the ME turquoise module were considered differentially expressed to the greatest extent. (The lowest p value). Additionally, the profiles obtained as mentioned above were included in the intersection to obtain the significantly differentially expressed IRGs. Furthermore, by leveraging univariate Cox proportional hazard regression, prognosis-related immune genes in the training cohort were screened out with the help of the R packages ‘survival’ and ‘survminer’, with the screening criterion set to a p value < 0.05. Moreover, the IRG-related prognostic model was constructed by a multivariate Cox proportional hazards model based on prognosis-related immune genes. The risk score was calculated using a linear combination of the Cox coefficient and gene expression as follows:

$$Risk\,score=\sum\nolimits_{i=\mathit1}^n\left(Expi^{\,\ast}\,Coei\right),$$

where N, Expi, and Coei represent the gene number, level of gene expression, and coefficient value, respectively. The median risk score was considered the cutoff value to divide all LUAD patients into high-risk and low-risk groups. In the model, the risk score reflected the prognosis of LUAD patients: a higher score indicated a worse prognosis. TCGA data were selected as the training cohort, while two GEO datasets were selected as the test cohort. Finally, to assess the prognostic prediction value of the model, both the training cohort and the test cohorts were enrolled in the time-dependent survival curve analysis by using the R package ‘timeROC’.

### Validation of the prognostic model

To evaluate the accuracy of this prognostic model, time-dependent ROC analysis was leveraged, and comparisons with other models were further performed. Moreover, the prognostic value of the IRG risk model was evaluated by leveraging both univariate and multivariate analyses of prognostic factors using Cox proportional hazards regression. Age and risk scores were treated as ordinal variables. Gender was coded as male (1) and female (0), and stage was treated as an ordinal variable, coded as stage I (1), stage II (2), stage III (3), and stage IV (4). Variables with a p value < 0.05 based on univariate analysis were further enrolled in multivariate analysis. Only variables with p values < 0.05 in both univariate and multivariate analyses were identified as independent prognostic factors. We constructed a nomogram to further explore the correlation between some clinicopathological characteristics and the prognostic model. Calibration curves were applied to appraise the consistency between the actual survival results and predictions.

### Pathway and enrichment analysis

To probe the significant biological processes of these differentially expressed IRGs, pathway and enrichment analyses were performed with the R package ‘clusterProfiler’. Gene Ontology (GO) and Kyoto Encyclopedia of Genes and Genomes (KEGG) enrichment analyses were performed. P-adjusted values < 0.05 were considered significant thresholds. With the help of the R package ‘ggplot2’, the top 30 terms or pathways were displayed.

To assess the functions associated with subtypes, gene set enrichment analysis (GSEA) was used by implementing the R package ‘clusterProfiler’ and ‘limma’ with the hallmark gene sets (h.all.v7.5.symbols.gmt) and the GO-BP subsets of the canonical pathway gene sets (c2.cp.go.v7.5.symbols.gmt).

### Analysis of immune cell characteristics

The proportions of the immune-related cells from each sample were calculated using the R package ‘CIBERSORT’. CIBERSORT [26] was used to analyze the relative expression levels of 547 genes in individual tissue samples according to their GEPs to predict the proportion of 22 types of TIICs in each tissue, including naive B cells, memory B cells, plasma cells, CD8 + T cells, naive CD4 + T cells, CD4 + resting memory T cells, CD4 + memory-activated T cells, follicular helper T cells, regulatory T cells, γδ T cells, resting natural killer cells, activated natural killer cells, monocytes, M0 macrophages (M0), M1 macrophages (M1), M2 macrophages (M2), resting dendritic cells, activated dendritic cells, resting mast cells, activated mast cells, eosinophils, and neutrophils. A *p* value < 0.05 and 100 × permutation count was considered significant for subsequent analysis. Additionally, the differences in the distribution of immune cells in the high- and low-risk groups were compared. Then, survival curves were completed based on immune-related cells. Finally, we explored the relationship between the risk score and immune cell infiltration in the tumor microenvironment.

### TMB analysis

Based on data collected from TCGA, we calculated the TMB of each patient (mutations per million bases) using Strawberry Perl. Then, LUAD patients’ somatic variant data were analyzed and visualized using the package ‘maftools’. The association between TMB and prognostic model risk score was further analyzed.

### Clinical utility of this model

The relationships between our model and the clinicopathologic features (age, sex, pathological stage, T stage, M stage, and N stage) were assessed to evaluate the prediction ability of the model in LUAD patients. All patients were divided into two groups (high-risk group and low-risk group) according to the risk score obtained previously. Age was treated as a categorical variable (< = 65 and > 65), sex was coded as female and male, and pathological stage, T stage, N stage, and M stage were treated as ordinal variables.

### Single-cell analysis

Single-cell sequencing data were downloaded from the GEO database (GSE203360). Single-cell analysis was conducted based on Seruatv4.1.1. First, we filtered out the genes with low expression in cells. Then, the filtered expression matrix was normalized by the NormalizeData function with the default parameters. Moreover, the top 3000 genes with the highest variations were obtained using the FindVariableFeatures function with the default ‘vst’ method. Principal component analysis (PCA) was further conducted based on the scaled variable gene expression. The nearest neighbor graph was constructed using the FindClusters function, and several cell clusters were identified based on the first ten principal components. Uniform Manifold Approximation and Projection (UMAP) was used to exhibit various cells in low dimensions. Finally, the differentially expressed genes were obtained using the FindMarkers function by Wilcoxon rank-sum test with the criteria that the │logFC│ between the two groups exceeds 0.25 and the gene expression difference between the two groups is statistically significant. The results are displayed in violin, bubble, and volcano plots.

### Cell culture and transfection

Lung cancer cell lines including A549, H1299, and H1650 cells was purchased from ATCC. The normal human bronchial epithelial (BEAS-2B) cell line was also purchased from ATCC. All the cells were cultured in the 1640 medium (Gibco, USA) with 10% FBS (HyClone Sera, USA) and 1% penicillin‐streptomycin (Sangon Biotech, China) in an atmosphere of 5% CO2 at 37 °C.The BIRC5 shRNA expression vector and scrambled shRNA nontarget control were obtained from Genewiz. Plasmids (Genewiz, China) were transfected through Lipofectamine 3000 (Thermo Scientific, USA) following the instructions from the manufacturer’s protocols.

### RNA Extraction and Quantitative real-time polymerase chain reaction (qRT-PCR)

TRIzol Reagent (Thermo Scientific, USA) was used to extract RNA following the manufacturer’s protocol. RevertAid First Strand cDNA Synthesis Kit (Thermo Scientific, USA) was chosen to conduct the reverse transcription experiment. First-strand cDNA was generated during the procedure. To detect the expression of genes, qRT-PCR following the SYBR protocol was carried out on a Roche lightcycler 480 PCR System by using ChamQ SYBR qPCR Master Mix (Vazyme, China). The following primers were used in PCR: BIRC5, forward, 5'-TGC CTGGCAGCCCTTTC-3' and reverse, 5'-CCTCCAAGAAGGGCCAGTTC-3’; GAPDH, forward, 5'-GAGTCAACGGATTTGGTCGT-3', and reverse, 5'-TTGATTTTGGAGGGATCTCG-3'.

### Western blotting analysis

RIPA Buffer (Thermo Scientific, USA) with Protease Inhibitor Cocktail (Sangon Biotech, China) was used to extract the proteins from cells, and the concentration of cell lysates was detected by BCA Protein Assay Kit (Sangon Biotech, China). The absorbance at 570 nm was measured (BioTek Epoch, USA). Equal quantities of proteins were separated by 12.5% sulfate–polyacrylamide gel electrophoresis (SDS-PAGE), and then the proteins were transferred to 0.2 μm NC membranes (GE whatman, USA). Nonspecific antigens on the membranes were blocked by incubating the membranes in 5% skim milk. Primary antibodies were incubated with the membranes at 4 °C overnight. The HRP-conjugated secondary antibody was applied to the membranes and incubated for two hours. The signals of each washed membrane were detected by electrochemiluminescence. All the antibodies were purchased from ABclonal.

### Cell Counting Kit-8 (CCK-8) Assay

A total of 2*10^3^ cells in each plate were incubated under the conditions mentioned in the cell culture section in 96-well plates. At 0 h, 24 h, 48 h, 72 h and 96 h, 10 μl CCK-8 solution mixed (Sangon Biotech, China) with 90 μl RPMI 1640 medium was added to each plate and incubated for 2 h at 37 °C. The absorbance at 450 nm was measured (BioTek Epoch, USA).

### Clone formation assay and EdU

Five hundred cells were inoculated in 6-well plates. The inoculated cells were then cultured in medium containing 10% FBS for fourteen days. Colonies were fixed with 4% paraformaldehyde for 60 min at room temperature and then stained with crystal violet for 60 min at room temperature. The number of colonies of each group was counted and statistically analyzed. EdU staining was carried out using the EdU kit (Beyotime Biotechnology, China) according to the manufacturer’s instructions. EdU-positive rate = EdU-positive cell count/cell count *100%.

### Statistical analysis

Statistical analysis was performed with R 4.1.0 (https://www.R-project.org). The differences in continuous variables between the two groups were measured by independent t tests or nonparametric Wilcoxon tests. We used chi-square tests to calculate categorical variables. The Kaplan–Meier method and log-rank test were applied for survival analysis. Univariate and multivariate Cox regression analyses were performed to explore the correlation between clinicopathologic features and our prognostic model.

## Results

### Development and Evaluation of Immune-Related Prognostic Model Based on the TCGA Dataset

**Fig. 1:**
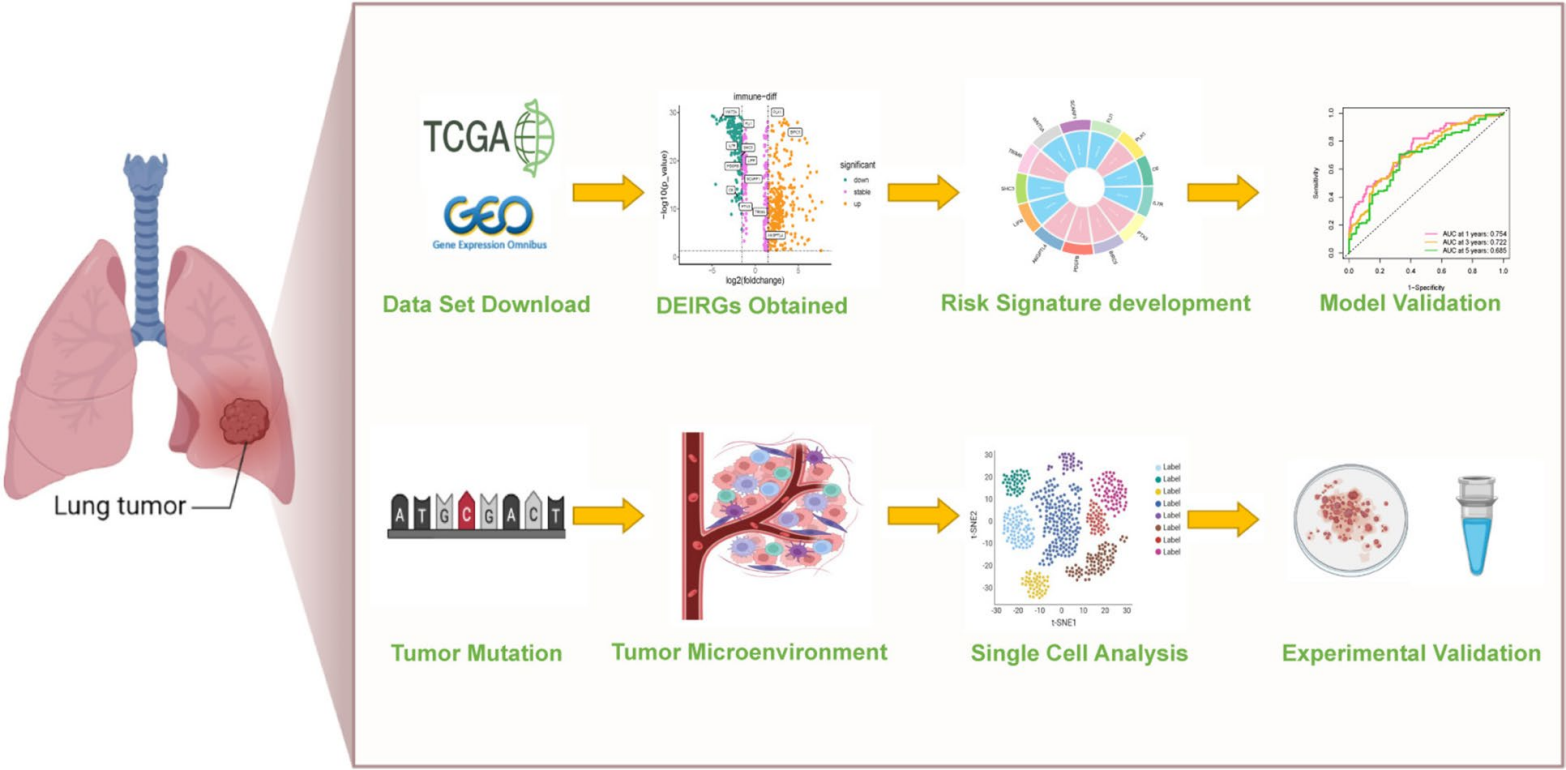
The workflow of the present study

The detailed flow chart for the prognostic model construction was exhibited in Fig. 1.

### The characteristics of patients

RNA-sequencing profiles and clinical data of 486 LUAD patients were downloaded from the TCGA-LUAD dataset (https://portal.gdc.cancer.gov/), constituting the training cohort. Moreover, the GEO dataset (https://www.ncbi.nlm.nih.gov/geo/) was leveraged to construct the test cohorts, one of which consisted of data from 398 LUAD patients (GSE72094) and the other consisted of data from 114 LUAD patients (GSE26939). Then, the training cohort was used to develop the prognosis risk model, while the test cohorts were applied for validation. The clinical characteristics of both the training cohort and the test cohorts are summarized in Supplementary Table S1.

### Identification of differentially expressed immune-related genes (DEIRGs) and functional enrichment analysis

**Fig. 2:**
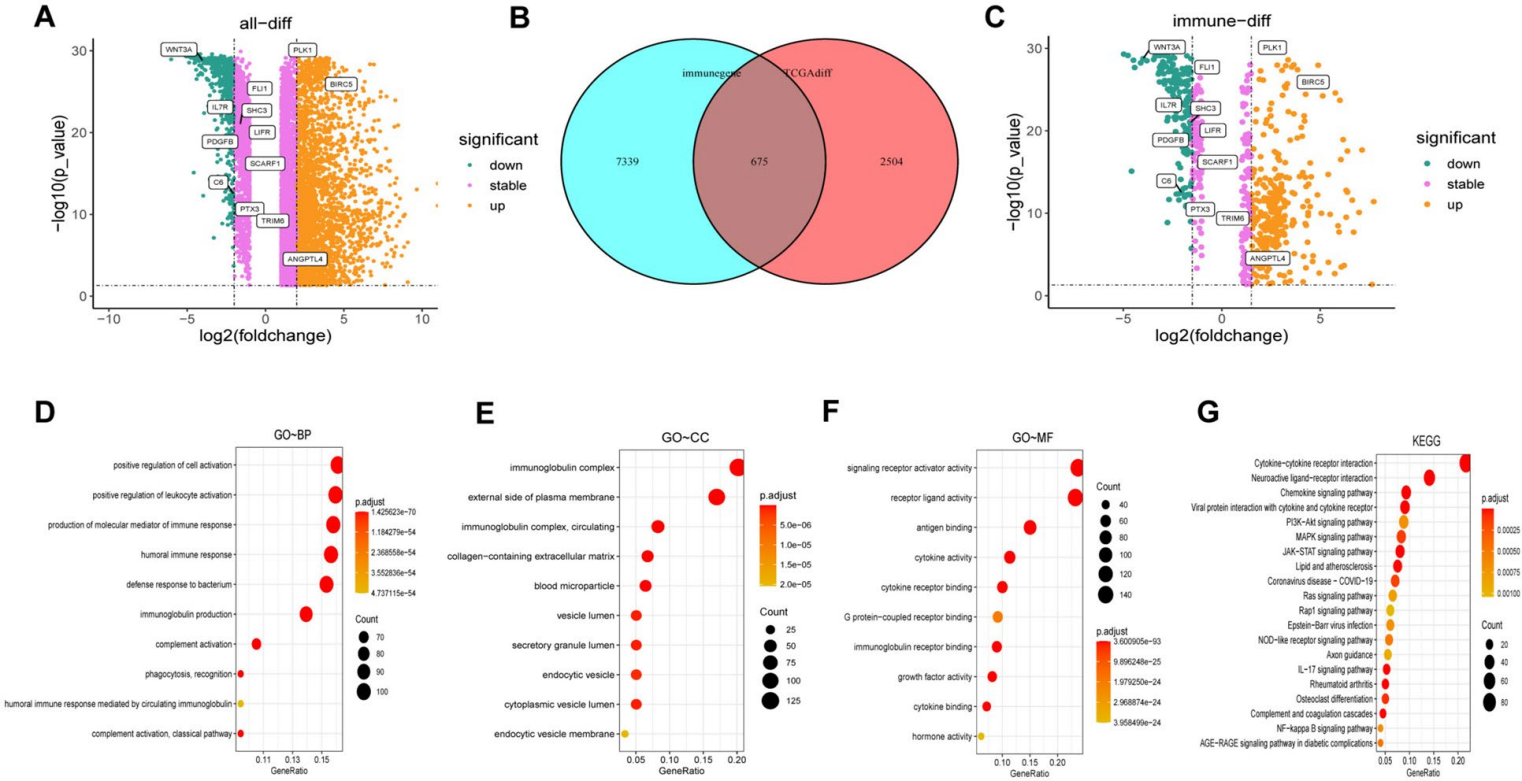
Identification of differentially expressed (DE) immune-related genes (DEIRGs). **A** Volcano plot of the DE genes. **B** Interaction between immune-related genes and differentially expressed genes. **C** Volcano plot of DEIRGs. **D**-**F** Top 10 terms for Gene Ontology (GO) analysis of the DEIRGs. **G** Top 20 terms for Kyoto Encyclopedia of Genes and Genomes (KEGG) analysis of the DEIRGs [27–29].

By comparing LUAD tissues with normal tissues, 8013 DE genes were identified, including 1912 downregulated and 6101 upregulated genes. Based on the two dimensions of -log10FDR and log2FC, the distribution of all DE genes is represented by a volcano map in Fig. 2A, thirteen of which are labeled and represent the genes involved in the prognostic model (constructed below). Additionally, 2660 immune genes were collected from the IMMPORT and InnateDB databases. Based on DE and immune-related genes, 675 DE immune genes, consisting of 260 downregulated and 415 upregulated genes, were obtained (Fig. 2B). Similarly, the DEIRGs (differentially expressed immune-related genes) are shown in Fig. 2C.

Functional analysis of DE genes was performed based on Kyoto Encyclopedia of Genes and Genomes (KEGG) and Gene Ontology (GO) functional enrichment analysis. As shown in Fig. 2D-F, the top 10 GO terms associated with biological process (BP), cellular component (CC), and molecular function (MF) are displayed. The DEIRGs were found to be significantly enriched in positive regulation of cell activation, immunoglobulin complex, and signaling receptor activator activity. Interestingly, in terms of BP, the DEIRGs dramatically correlated with immune-related functions, such as positive regulation of leukocyte activation and production of molecular mediators of the immune response. Likewise, the top 20 enriched pathways are represented in Fig. 2G, the results of which demonstrated that DE immune genes were significantly enriched in the cytokine − cytokine receptor interaction, neuroactive ligand − receptor interaction, and chemokine signaling pathways.

### Construction of the prognostic model based on DEIRGs

**Fig. 3:**
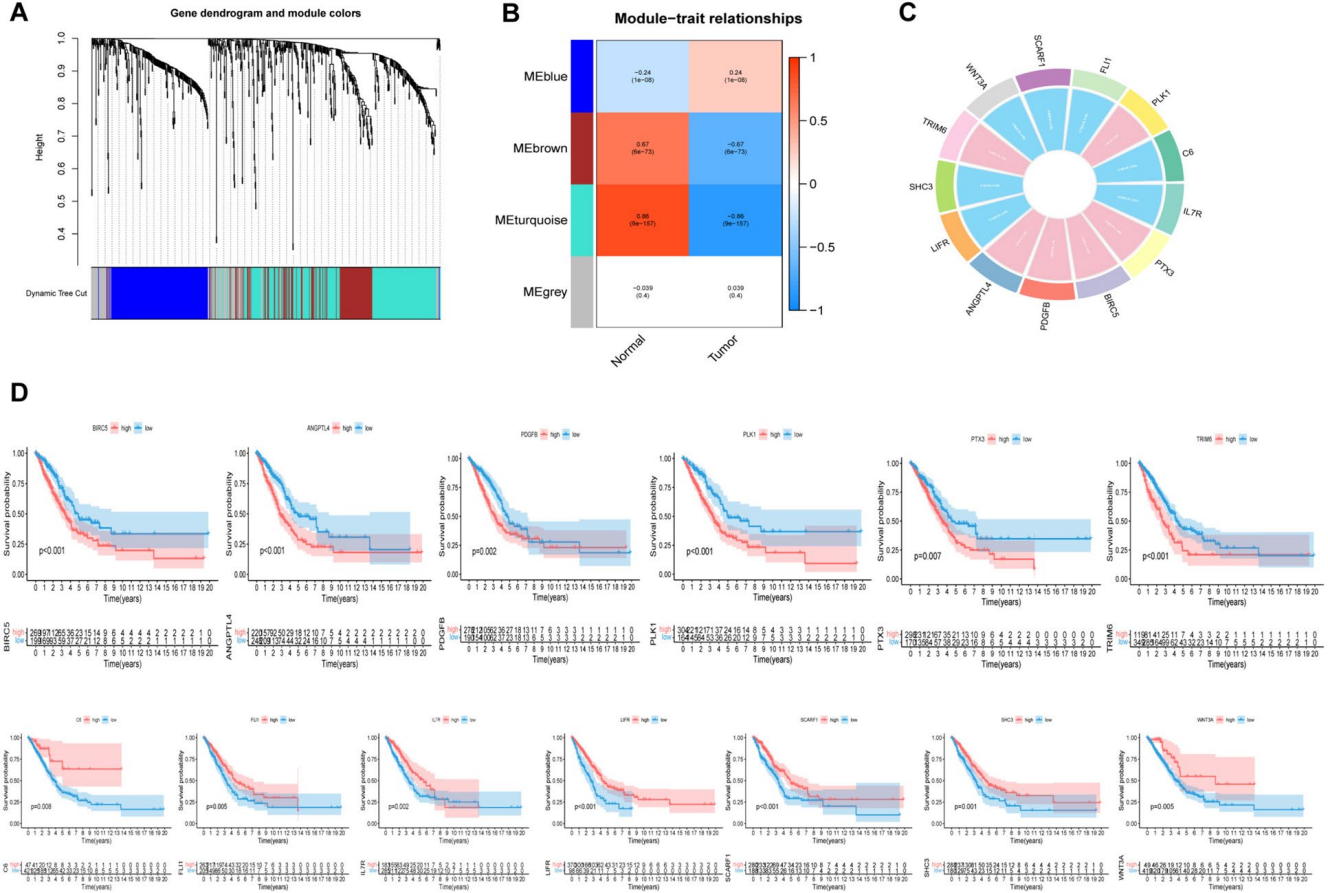
Weighted correlation network (WGCNA) analysis and prognostic model construction. **A**, **B** Cluster dendrogram and module assignment for modules based on WGCNA. Genes cluster dendrogram drawn by use of a dissimilarity measure (1-TOM). The colored horizontal bar represents the modules, lying below the dendrogram. A total of 675 immune related genes were assigned to one of 4 modules including the turquoise module. **C** Circle plot displayed the result of univariate analysis (**D**) Kaplan–Meier survival curves of 13 immune-related hub genes obtained by univariate Cox regression analysis

Using WGCNA, DEIRGs were grouped into modules to aggregate genes with similar traits (Fig. 3A); then, the ‘MEturquoise’ module with the highest correlation with tumors (Cor = -0.86, *P* = 9e-157) was identified as a tumor-specific module (Fig. 3B). Then, after interacting the transcriptome sequencing data collected from the TCGA database and GEO database (GSE72094) and the genes involved in the ‘MEturquoise’ module (based on WGCNA), the immune-related genes coexpressed in the TCGA and GEO databases that were differentially expressed to a great degree between lung adenocarcinoma samples and normal samples were obtained. Based on the training cohort, 56 immune-related genes (that were possible prognostic genes) were obtained by univariate Cox analysis (Figure S1). These genes were then enrolled in multivariate Cox analysis to acquire 13 optimal immune genes (as shown in Fig. 3C) based the constructed prognosis risk model. The risk model formula is presented in the materials section, while the risk coefficient is summarized in Supplementary Table S2*.* Finally, a survival analysis of these genes was conducted on the condition that the median risk point was considered the cutoff value to divide all LUAD patients into high-risk and low-risk groups. As depicted in Fig. 3D, *ANGPTL4, BIRC5, PDGFB, PLK1, PTX3,* and *TRIM6* serve as risk genes, while *C6, FLI1, IL7R, LIFR, SCARF1, SHC3,* and *WNT3A* function as protective genes (survival analysis of genes in GSE72094 is presented in Figure S2).

### Validation of the prognostic model

**Fig. 4:**
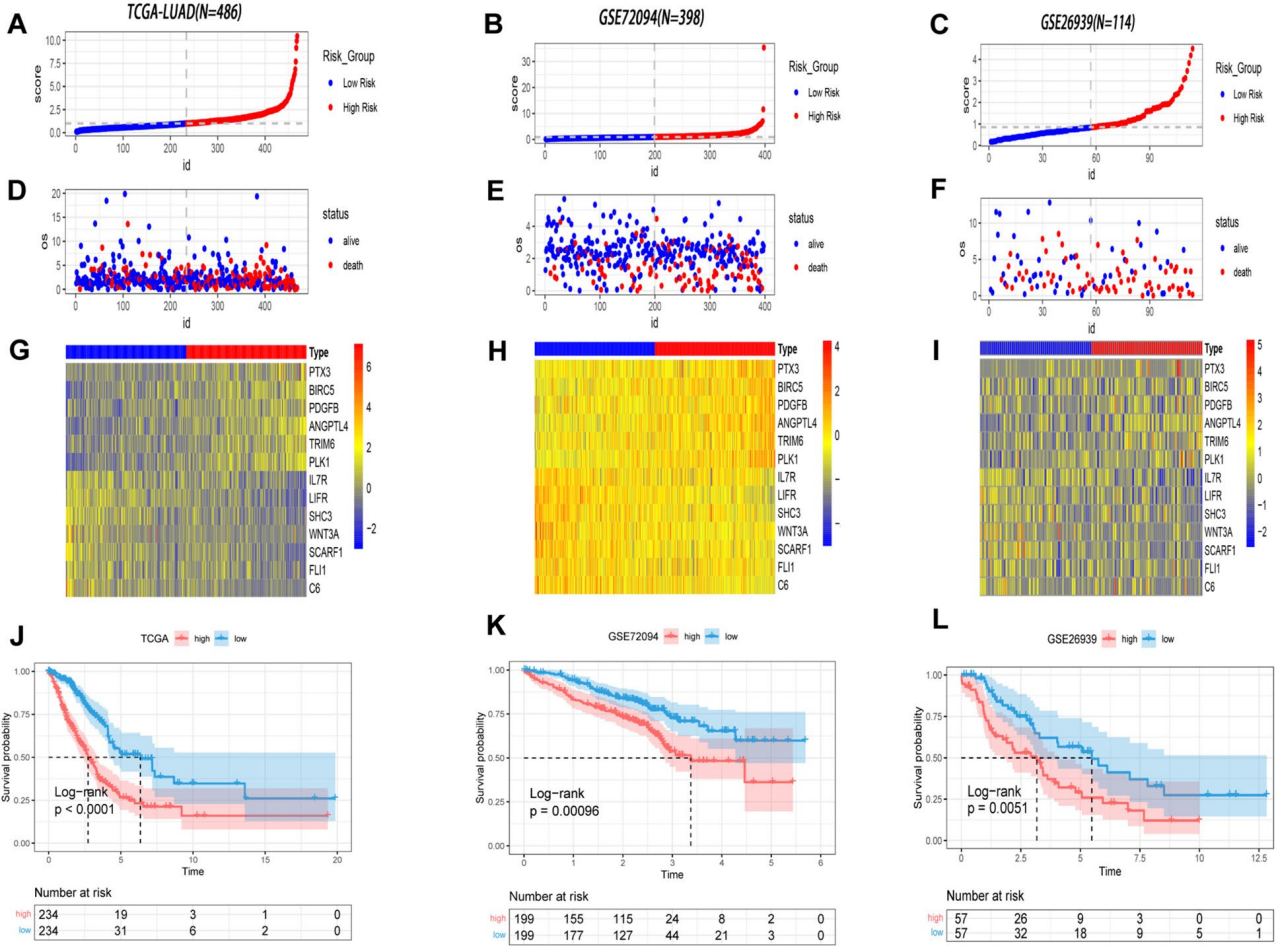
Correlations between risk score and prognosis of LUAD patients. **A**-**C** patient risk score distribution, **D**-**F** scatter diagram of patient survival status, and **G**-**I** expression pattern of prognostic genes respectively of training cohort and two test cohorts. **J**-**L** KM survival curves of TCGA training cohort and GEO validation cohorts based on high- and-low risk groups

Compared with the low-risk group, the high-risk group had a significantly higher proportion of deaths and shorter survival times in both the training cohort and test cohort (Fig. 4A-F). Moreover, as depicted in the heatmaps (Fig. 4G-I), *C6, FLI1, IL7R, LIFR, SCARF1, SHC3,* and *WNT3A* had higher expression levels in the low-risk group; in contrast, *ANGPTL4, BIRC5, PDGFB, PLK1, PTX3,* and *TRIM6* had higher expression levels in the high-risk group*,* consistent with the identification presented before*.* Then, both the training cohort and test cohorts were enrolled in the survival curve analysis, as shown in Fig. 4J-L. A significant difference in survival curves was found between the high-risk group and the low-risk group, preliminarily reflecting the reliability of our model.

**Fig. 5:**
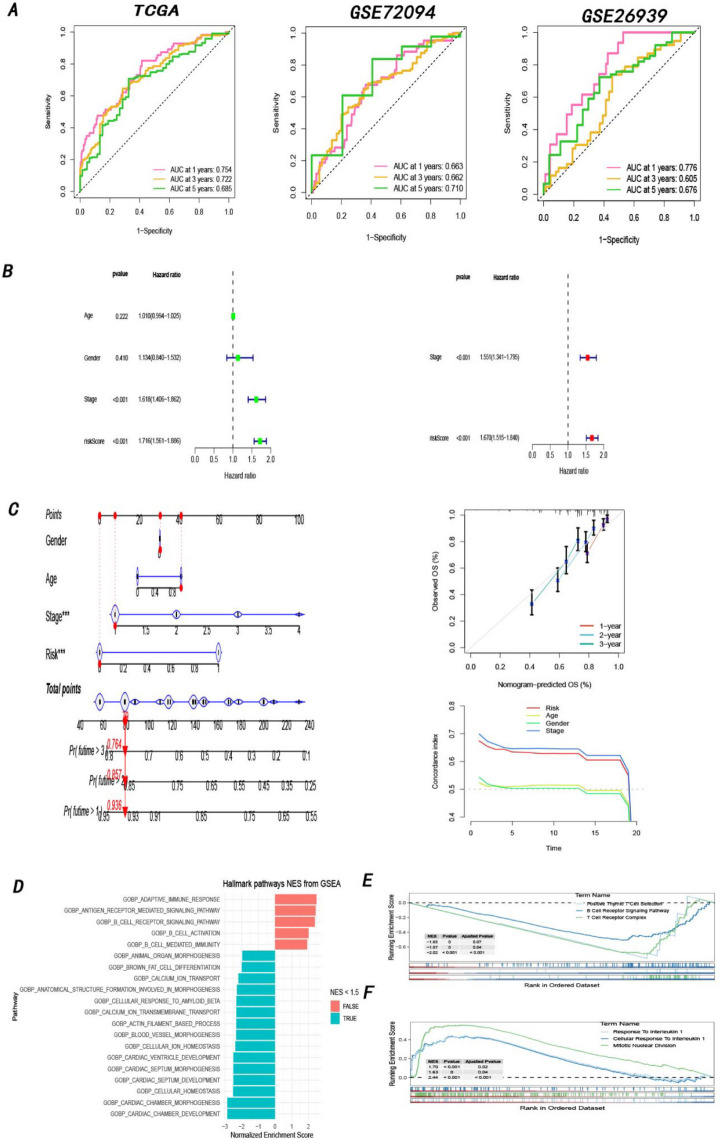
Validation of the prognostic model (**A**) Time ROC curves of the training cohort and test cohorts. **B** Multivariate as well as Univariate Cox regression analysis. **C** Nomogram consisted of several clinicopathologic features, and consistency between predicted and actual survival rates by calibration and C-index. **D** GSEA analysis of DEIRGs with the hallmark gene sets (h.all.v7.5.symbols.gmt). **E**–**F** GSEA analysis based on high-and-low risk groups with the GO-BP subsets of the canonical pathway gene sets (c2.cp.go.v7.5.symbols.gmt)

Next, the area under the curve (AUC) in the time-dependent ROC analysis was further analyzed in both the training cohort and test cohorts to predict the performance of the prognostic model. As shown in Fig. 5A-C, the AUC of the risk score model was 0.754 at 1 year, 0.721 at 3 years, and 0.681 at 5 years in TCGA; 0.663 at 1 year, 0.662 at 3 years, and 0.710 at 5 years in GSE72094; and 0.776 at 1 year, 0.605 at 3 years, and 0.676 at 5 years in GSE26939, revealing the excellent specificity and sensitivity of the prognostic risk score. Furthermore, univariate Cox analysis illuminated that stage (hazard ratio: 1.618, 95% confidence interval: 1.406–1.862) and the prognostic model (hazard ratio: 1.716, 95% confidence interval: 1.561–1.886) were independent risk factors for the prognosis of LUAD patients. Likewise, multivariate Cox analysis confirmed that both stage (hazard ratio: 1.551, 95% confidence interval: 1.341–1.795) and the prognostic model (hazard ratio: 1.670, 95% confidence interval: 1.515–1.840) were significantly related to the prognosis of LUAD patients (Fig. 5B). In addition, a nomogram integrating the prognostic model and other clinical characteristics was established for quantitative prediction. Then, the performance of the nomogram was proven to be robust by the C-index and calibration curve (Fig. 5C). These findings collectively validated that the prognostic model could function as a reliable independent prognostic factor for patients with LUAD.

### GSEA (Gene Set Enrichment Analysis)

The DEIRGs in the TCGA database were subjected to GSEA, and the immune-related pathways were found to be significantly correlated with the NES (normalized enrichment score) > 1.5 (Fig. 5D). Additionally, GSEA enrichment was performed between the high-risk group and the low-risk group. Notably, some immune-related pathways were activated in low-risk patients, including B Cell Receptor Signaling Pathway, Positive Thymic T Cell Selection, and T Cell Receptor Complex (Fig. 5E), while Response To Interleukin 1 was significantly enriched in high-risk group (Fig. 5F).

### Risk score and TMB (Tumor Mutation Burden)

**Fig. 6:**
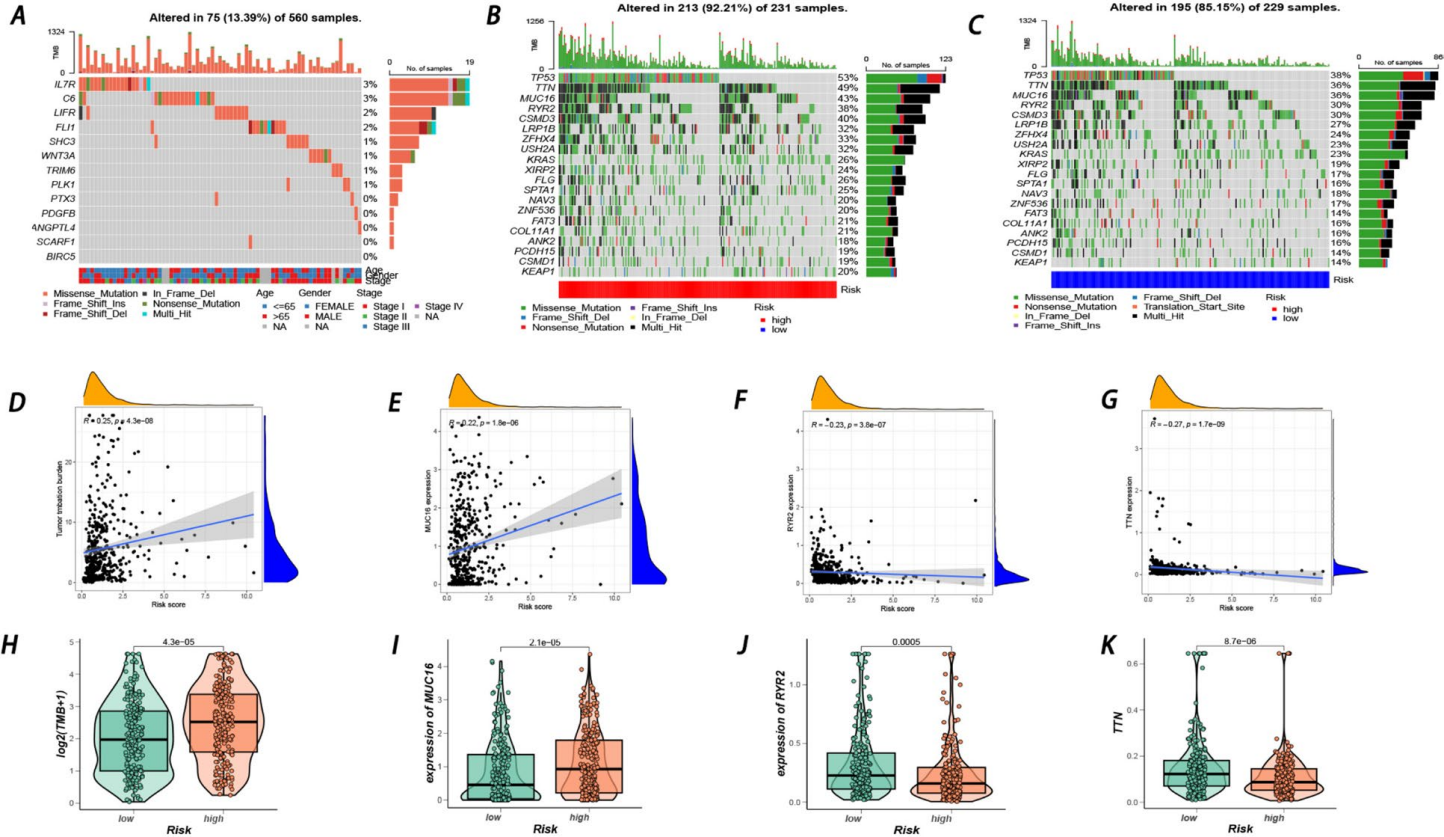
**A** Gene mutation of LUAD patients based on 13 prognostic genes. **B** Gene mutation of patients within the high-risk group. **C** Gene mutation of patients within the low-risk group. **D**, **H** Correlations between TMB and risk score. **E**, **F**, **G**, **I**, **J**, **K** Correlations between several hypervariable genes and risk score

Subsequently, the gene mutations of each LUAD patient were analyzed. Figure 6A reflects the mutation context of genes in the prognostic model (*ANGPTL4, BIRC5, PDGFB, PLK1, PTX3, TRIM6, C6, FLI1, IL7R, LIFR, SCARF1, SHC3,* and *WNT3A*). Then, the top 20 genes with the highest mutation frequencies (*TP53, TTN, MUC16, RYR2, CSMD3, LRP1B, ZFHX4, USH2A, KRAS, XIRP2, FLG, SPTA1, NAV3, ZNF536, FAT3, COL11A1, ANK2, PCDH15, CSMD1,* and *KEAP1*) were separately depicted in high-risk patients and low-risk patients (Fig. 6B-C). In addition, the TMB was higher in high-risk patients (R = 0.25) (Fig. 6D,H), indicating that the TMB might contribute to tumor initiation and progression. Furthermore, of the top 10 genes with the highest mutation frequencies, the expression of MUC16 was found to be positively correlated with the TMB, while the expression of RYR2 and TTN was identified to be negatively related to the TMB (Fig. 6E-G,I-K).

### Associations between the prognostic model and clinical characteristics in LUAD Patients

**Fig. 7:**
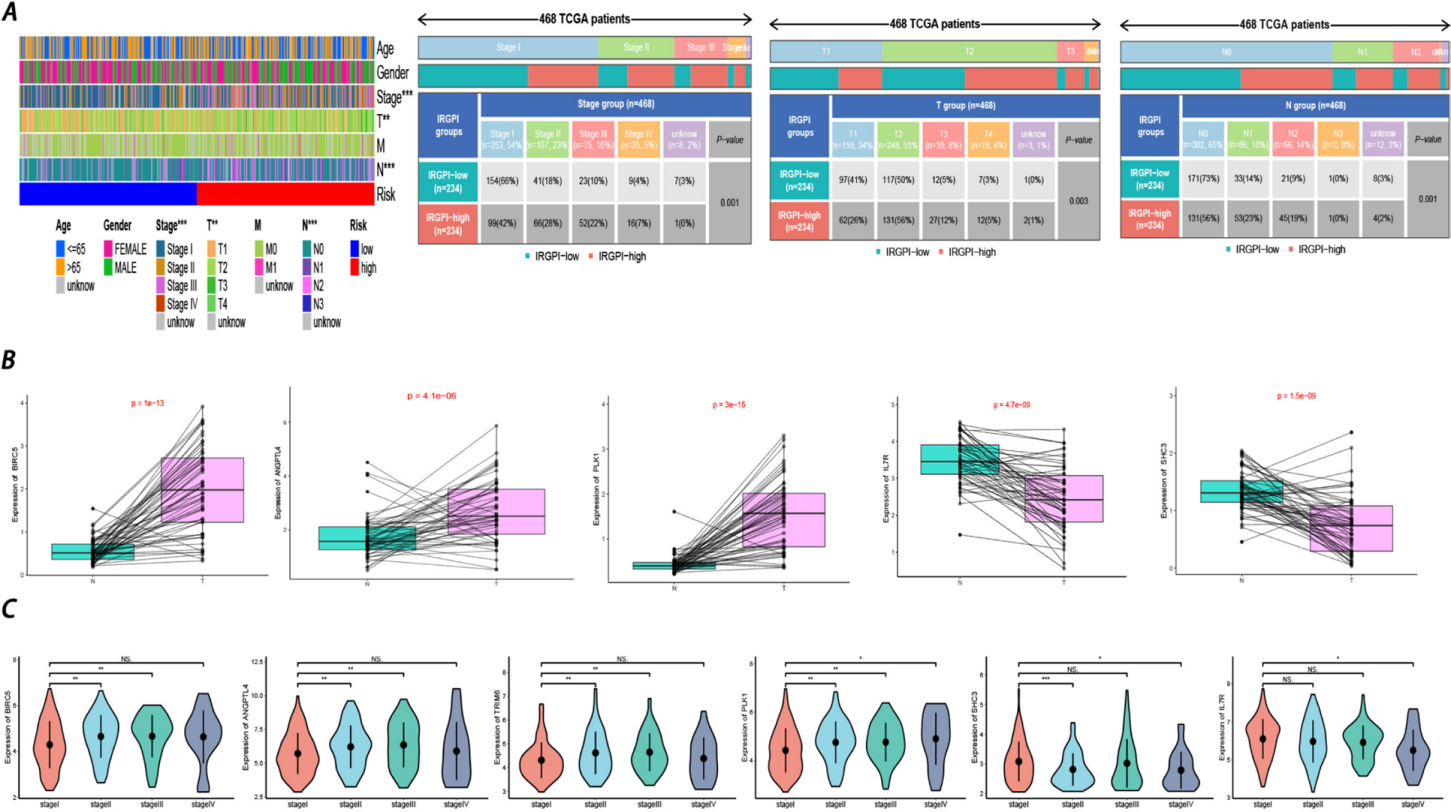
**A** The heatmaps reflected the associations between prognostic-risk model and clinicopathological characteristics in the training cohort. (*p-value <  = 0.05; *p*-value <  = 0.01; ***p-value <  = 0.001) **B** Expression level difference of several prognostic genes between tumor samples and the paired normal ones. **C** Correlations between expression level of the prognostic genes and clinical-stage

After analyzing the clinical characteristics (age, sex, stage, T stage, N stage, and M stage) of the high-risk and low-risk patients, stage, T stage, and N stage were found to be remarkably correlated with the prognostic model. Notably, patients in the high-risk group tended to have more severe clinical stages (Fig. 7A). Furthermore, we analyzed the relationship between prognostic genes and characteristics. Among the 13 prognostic genes, the expression of *BIRC5, ANGPTL4*, and *PLK1* was significantly higher in tumor samples, while the expression of *IL7R* and *SHC3* was higher in normal samples. In addition, higher *BIRC5, ANGPTL4, TRIM6*, and *PLK1* gene expression correlated with a more severe clinical stage for patients. In contrast, the expression of *SHC3* and *IL7R* was negatively associated with clinical stage. In summary, *BIRC5, ANGPTL4*, and *PLK1* might act during the origin and progression of LUAD, while *IL7R* and *SHC3* might serve as protective factors (Fig. 7B-C).

### Immune cell proportions between low-and-high risk scores in LUAD patients

**Fig. 8:**
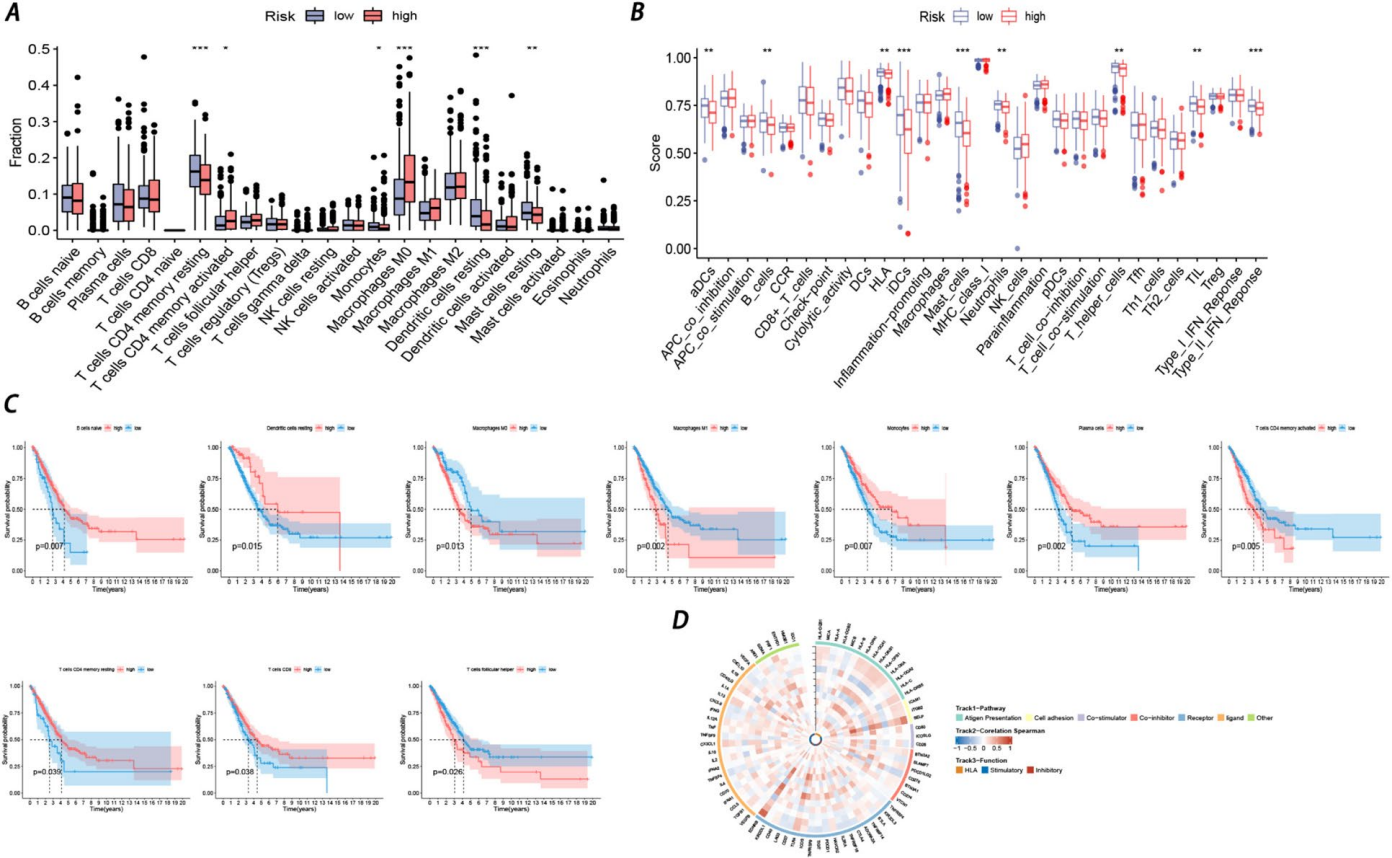
Immune cell infiltrations analysis (**A**) Infiltration abundance of 22 immune cells based on high-and-low risk groups. **B** Infiltration abundance of immune-related functions based on high-and-low risk groups. Blue represents the low-risk group and the red represents the low-risk group. The horizontal line represents the median, and the top and bottom of the box are the 75th and 25th percentiles (quartile intervals), respectively. The Wilcoxon test was used to evaluate the differences between the two groups (*P < 0.05, **P < 0.01, ***P < 0.001, ****P < 0.0001). **C** KM survival curves of immune cells based on high-and-low risk groups. **D** The ring heatmap showed the associations between the prognostic genes and 75 immune-related genes

With the CIBERSORT algorithm, 22 immune cell types were chosen in each LUAD sample and compared between the low- and high-risk groups. The proportions of 22 immune cells as well as immune-related functions are depicted in Fig. 8A-B. Compared with the low-risk group, the high-risk group revealed increasing proportions of activated memory CD4 T cells and M0 macrophages. In contrast, resting memory CD4 T cells, monocytes, resting dendritic cells, and resting mast cells accounted for remarkable proportions in the low-risk group compared with the high-risk group. Interestingly, in terms of immune-related functions, aDCs, B_cells, HLA, iDCs, mast_cells, neutrophils, T_helper_cells, TIL, and Type_II_IFN_Response overwhelmingly gained higher scores in the low-risk group. Then, 22 types of immune cells were subjected to survival analysis. Figure 8C shows that patients with high proportions of macrophage Mos, macrophage Mos, activated memory CD4 T cells, and follicular helper T cells tended to have short survival times. Conversely, high proportions of naive B cells, resting dendritic cells, monocytes, plasma cells, resting memory CD4 T cells, and CD8 T cells were remarkably correlated with long survival time (K-M analysis of immune-related functions is presented in Figure S3). Moreover, Fig. 8D shows the correlation between the 13 genes in the prognostic model and the 75 immune-related genes.

### Landscape of immune and stromal cell infiltrations in the low- and high-risk groups and therapeutic benefit prediction

**Fig. 9:**
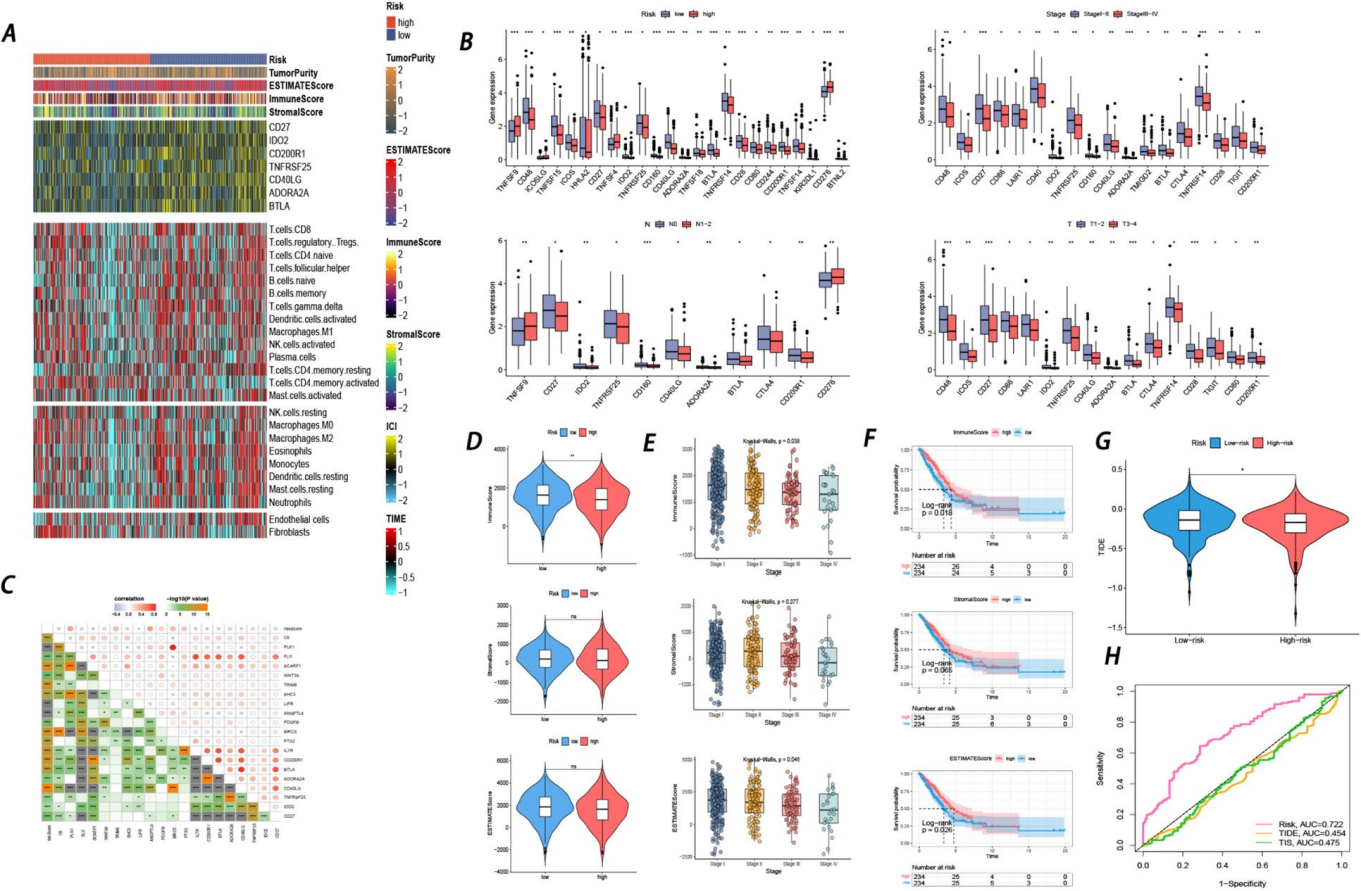
Tumor microenvironment and immunotherapy response (**A**) Multialgorithm analytical results on immune cells of tumor microenvironment (TME) in LUAD, including existing data from platform TIMER and MCP-counter. The top-bars show the distribution of TME-related scores. **B** Correlations between expression level of immune checkpoints and the risk score along with vital clinicopathological features. **C** The heatmap combined with bubble diagram demonstrated the correlations between prognostic genes and immune checkpoints along with the risk score. **D** Relationships between risk score and stromal score, immune score, as well as estimate score. **E** Associations between clinical-stage and stromal score, immune score, as well as estimate score. **F** KM survival curves based on stromal score, immune score, as well as estimate score. **G** Immunotherapy response prediction by TIDE. (H) Comparison between the prognosis prediction efficacy of our prognostic model and that of other models

After exploring the landscape of immune and stromal cell infiltrations in the low- and high-risk groups, Fig. 9A illustrates that patients in the high-risk group shared higher proportions of immune and stromal cell infiltrations than those in the low-risk group. In addition, the expression of 48 immune checkpoints was investigated and compared between the high- and low-risk groups. The relationship between their expression and patients’ clinical characteristics was further probed. In total, 6 checkpoints (*CD27, IDO2, CD200R1, TNFRSF25, CD40LG, ADORA2A,* and *BTLA*) were obtained (Fig. 9B); the expression of these genes was not only obviously correlated with risk scores but also significantly correlated with clinical characteristics. In line with Fig. 9A, these 6 checkpoints were remarkably modulated in the low-risk group. Figure 9C shows the correlation between 13 prognostic genes, risk score, and 6 immune checkpoints. All 6 immune checkpoints were strongly linked with the prognostic genes and risk score.

Then, we also investigated connections between the ImmuneScore, StromalScore, ESTIMATEScore, risk score and clinical stage (Fig. 9D-E). The results demonstrated that a high ImmuneScore correlated with low-risk scores and favorable clinical stage, which was consistent with the K-M survival analysis (Fig. 9F). However, in terms of response to immunotherapy, patients in the high-risk group had a lower TIDE score (Fig. 9G), reflecting that patients in the high-risk group might respond better to immunotherapy. TMB differences between high- and low-risk groups could account for the contradiction. Finally, the area under the curve ROC analysis exhibited better prediction of our prognostic model compared with that of others, such as TIDE and TIS (Fig. 9H).

### Single-cell analysis of genes in the prognostic model

**Fig. 10:**
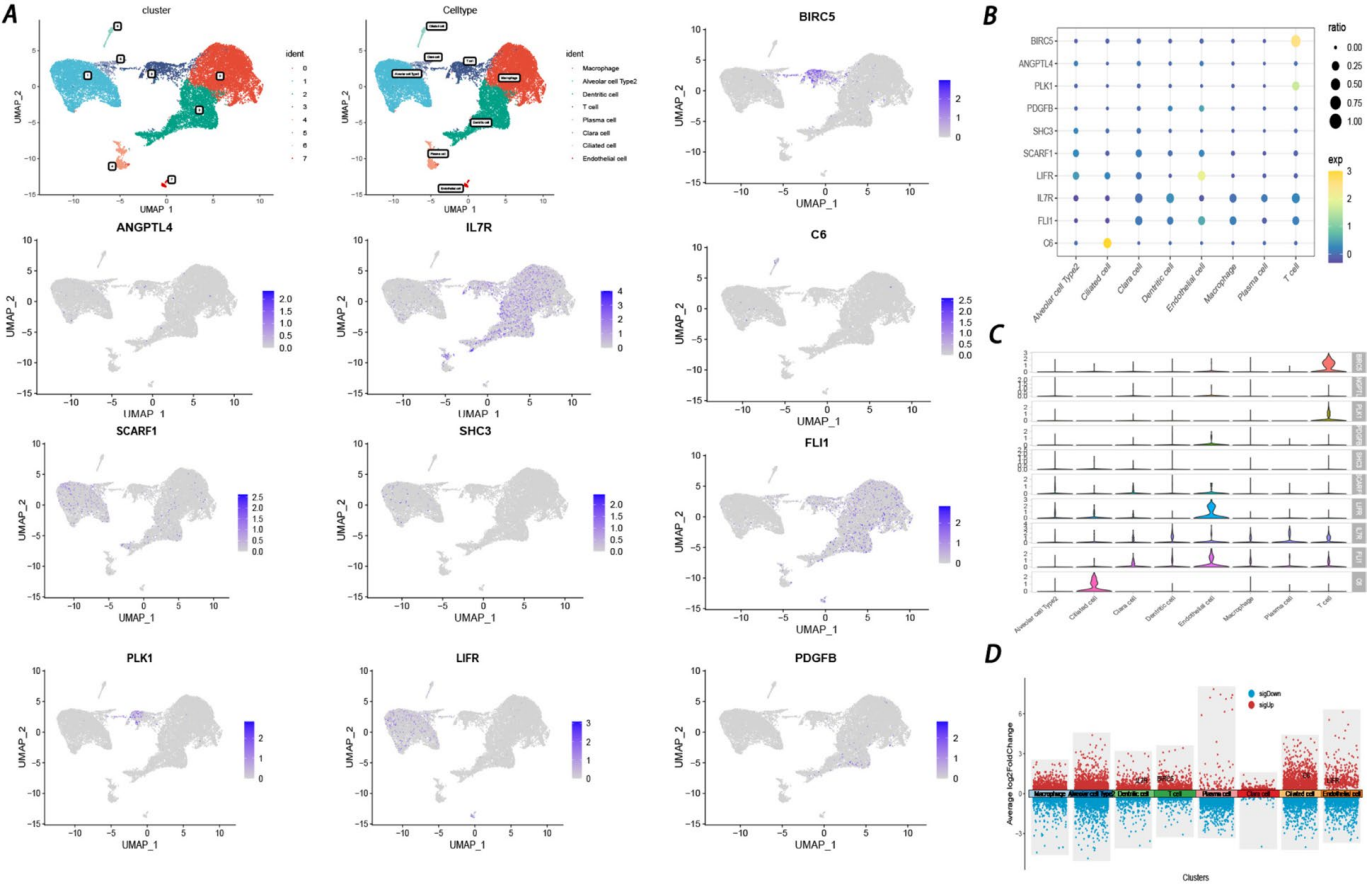
Evaluation of the prognostic genes’ expression level in different cells by single-cell sequencing (**A**) UMAP plot exhibited the prognostic genes’ expression level in different cells. Bubble diagram (**B**) violin plot (**C**) and volcano plot (**D**) showed these genes’ expression level in different cells

To validate the expression of prognostic genes in immune-related cells, single-cell analysis was performed with the GSE203360 dataset. A total of 19,363 cells were obtained and subjected to UMAP clustering analysis. Based on the expression of cell-type-annotation markers, all cells were clustered into eight major cell types, including macrophages, Alveolar cell Type 2, Dendritic cells, T cells, plasma cells, Clara cells, ciliated cells, and endothelial cells. Three genes (*PTX3, TRIM6, and WNT3A*) were not found in the dataset. In addition, the other 10 genes (*C6, FLI1, IL7R, LIFR, SCARF1, SHC3, ANGPTL4, BIRC5, PDGFB,* and *PLK1*) were presented in a UMAP plot with their microsatellite status (Fig. 10A). Interestingly, BIRC5 showed remarkable enrichment in T cells, while *ANGPTL4, C6, SHC3,* and *PDGFB* showed no enrichment. Additionally, *IL7R, SCARF1, FLI1, PLK1,* and *LIFR* exhibited slight enrichment in macrophages, Alveolar cell type 2, ciliated cells, T cells, and Alveolar cell type 2, respectively. Furthermore, according to bubble, violin, and volcano plots (Fig. 10B-D), BIRC5 was significantly enriched in T cells. Combined with the bulk RNA results obtained previously, BIRC5 was determined to be noticeably immune-related and significantly correlated with the prognosis of LUAD patients.

### Validation of the tumor-related role of BIRC5 in NSCLC

**Fig. 11:**
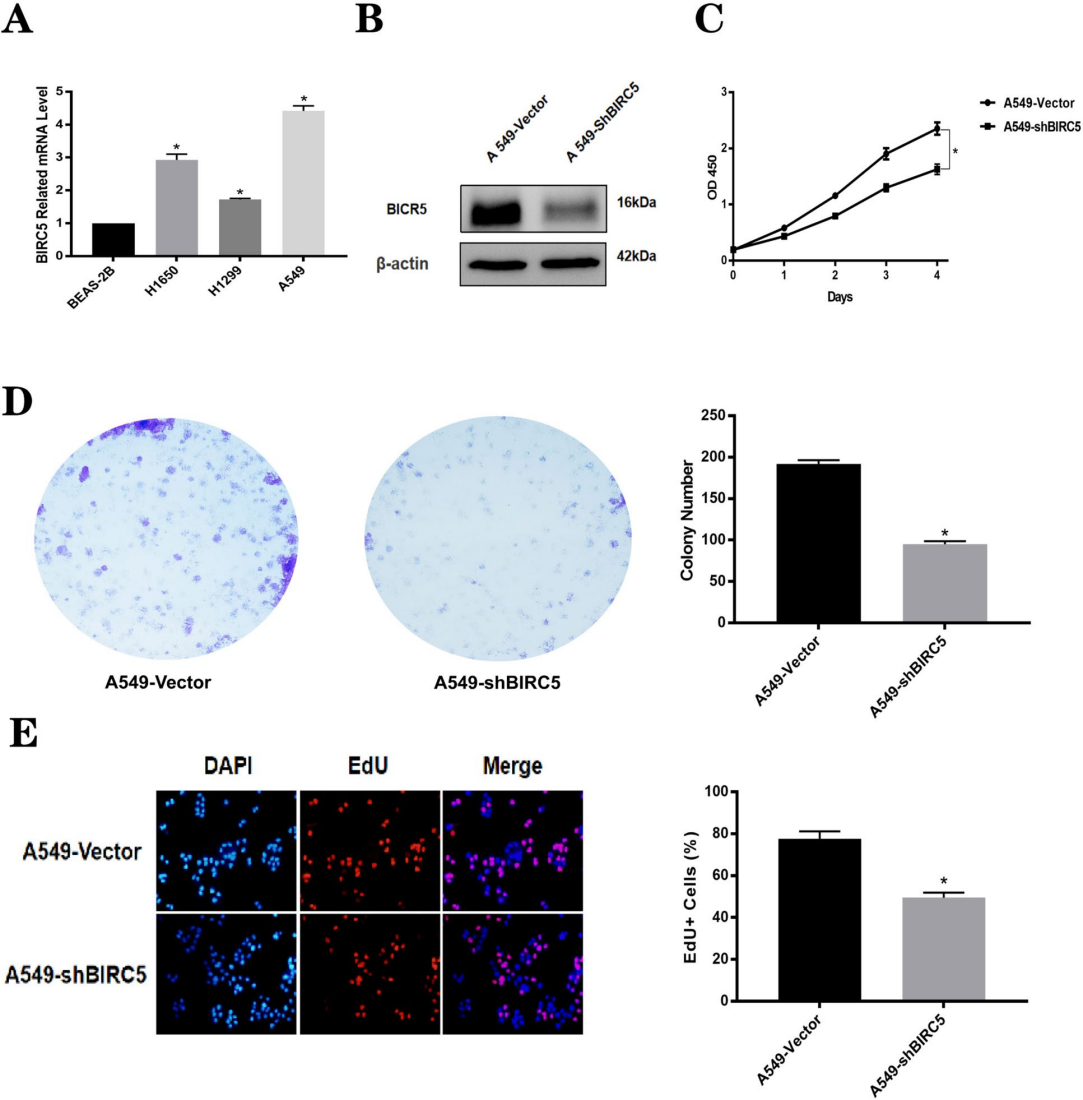
Validation of the oncogenic role of BIRC5 in lung adenocarcinoma cells. **A** qRT-PCR to evaluate the BIRC5-mRNA levels in different lung adenocarcinoma cell lines. **B** Western Blotting assay to confirm knockdown efficiency of sh-BIRC5 in A549 cells, using shRNA vector as the negative control.(The original, unprocessed versions of blots with membrane edges visible are included in the Supplementary Information file named ‘β-actin’ and ‘BIRC5’) (**C**) CCK-8 assay to test cell proliferation capacity after BIRC5-knockdown in A549 cells. **D** Colony formation assay was conducted to assess colony formation alteration after silencing BIRC5 in A549 cells. **E** Edu experiment was further performed to evaluate the different cell proliferation difference in A549 cells treated with BIRC5-shRNA or control cells

Finally, we conducted systematic cellular experiments to validate our in silico findings. After testing the mRNA level of BIRC5 in four different lung adenocarcinoma cell lines (BEAS-2B, A549, H1299, and H1650), we selected A549 cells for further experiments (Fig. 11A). The knockdown efficiency of shRNA targeting BIRC5 was confirmed by immunoblotting (Fig. 11B), and infected cells were then subjected to phenotype analyses. Accordingly, the CCK-8 assay showed that silencing BIRC5 significantly inhibited the viability of A549 cells compared to that of control cells (Fig. 11C). Similarly, colony formation assays demonstrated a decreased colony formation capacity after BIRC5 knockdown (Fig. 11D). Consistent with the CCK-8 and colony formation experiments, the EdU assay further confirmed that silencing BIRC5 remarkably suppressed lung cancer cell proliferation (Fig. 11E), highlighting its oncogenic role.

## Discussion

Recently, great progress has been made in immunotherapy, especially for non-small cell lung cancer (NSCLC), shedding novel light on the therapeutic strategy of patients diagnosed with NSCLC [30]. Nevertheless, many LUAD patients still suffer from this malignant tumor due to the low response rate [31]. The lack of precise therapeutic targets or limited knowledge of the TME might account for this dilemma [32]. The tumor microenvironment, consisting of not only diverse immune and stromal cells but also the factors they secrete, has been deemed to correlate with treatment efficacy and patient outcomes [33]. Under these circumstances, a prognostic model based on immune-related genes was constructed to help select patients for immunotherapy and discover potential biomarkers.

In our study, 675 DEIRGs were obtained between tumor and normal tissues based on TCGA and IMMPORT databases. Then, 56 immune-related genes were identified using univariate Cox regression analysis. Multivariate Cox regression analysis was applied to identify 13 key immune-related genes, calculate coefficients and construct the risk model. As expected, we found that patients in the high-risk group had shorter survival than those in the low-risk group. Subsequently, forest plots and a nomogram were constructed to evaluate the clinical applicability of the model. Plotting the ROC curve and survival curve established that our model had an excellent predictive effect. Furthermore, our model still performed well after external validation with two GEO datasets (GSE72094 and GSE26939). In addition, our model was remarkably correlated with prognostic malignant clinicopathologic characteristics (such as clinical stage, T stage, and N stage), further revealing its outstanding prognostic efficacy.

For the GSEA based on the DEIRGs, adaptive immune response, B-cell activation, and B-cell mediated immunity were significantly enriched, which could exert enormous influence on the tumor microenvironment, limiting tumor invasion to some extent [34, 35]. Then, all samples were subjected to GSEA based on the high- and low-risk groups. Interestingly, we found that mitotic nuclear division, which could lead to chromosomal instability and promote the migration of NSCLC [36], was remarkably enriched in the high-risk group. In addition, response to interleukin-1 and cellular response to interleukin-1 were also observed to be enriched. Besides, antigen receptor mediated signaling pathway was found enriched in high-risk group, which has been confirmed to mediate superior antitumor effects [37]. Recently, a paper revealed that the tumor response to cetuximab could be enhanced by increasing the levels of IL-1α [38]. In conclusion, patients in the high-risk group might have a worse prognosis but respond better to immunotherapy.

Tumors consist not only of cancer cells but also the tumor microenvironment, which consists of stromal cells (tumor-infiltrating immune cells and cancer-associated fibroblasts), the extracellular matrix, and various cytokines and metabolites. Representing most of the tumor mass, the TME actively participates in tumorigenesis [39]. The development and progression of cancer are accompanied by modifications in the adjacent stroma. Cancerous cells are capable of manipulating their microenvironment in a functional manner, by means of excreting diverse cytokines, chemokines, and other factors. As a consequence, a reprogramming of the neighboring cells is induced, allowing them to assume a decisive function in the sustenance and advancement of the neoplasm [40]. The TME can communicate with tumor cells, permitting them to proliferate and protecting them from apoptosis. Thus, the TME might play an essential role in therapeutic efficacy [41]. Under these circumstances, we comprehensively analyzed the TME with the ESTIMATE algorithm based on transcriptomic data. The immune score and stromal score represent the status of immune and stromal cell infiltration within the TME in LUAD. The results revealed that patients in the low-risk group shared remarkably higher immune scores than those in the high-risk group. In addition, the immune score was negatively correlated with clinical stage and positively associated with survival time, indicating that immune cells might function as protective factors, providing a favorable prognosis for patients diagnosed with LUAD. However, the stromal score was not found to be significantly linked to the risk score and clinical characteristics, which implied that stromal cells might not play a significant role in the tumorigenesis of our samples. Moreover, six immune subtypes of cancer could influence the prognosis by determining immune response patterns [42], which consist of C1 (wound healing), C2 (IFN-γ dominant), C3 (inflammatory), C4 (lymphocyte depleted), C5 (immunologically quiet) and C6 (TGF-γ dominant). The distribution of various immune subtypes between the high- and low-risk groups was analyzed by the chi-square test (Fig. S4). The results showed that the C1 and C2 subgroups accounted for more patients in the high-risk group (28% and 37%), while the low-risk group mainly correlated with the C3 subgroup. As reported, CD4^+^ T cells can function as tumor growth suppressors and induce cytolysis by secreting interferon-γ (IFN-γ) [43]. However, chronic inflammation can induce tumor progression, triggering treatment resistance [2]. To summarize, we can infer that patients in the high-risk group might respond well to immunotherapy based on immune subtype analysis.

Furthermore, to elucidate the TME immune landscape, we explored the infiltration status of 22 immune cells in LUAD. Consistent with previous results, most of the immune cells were enriched in the low-risk group, including resting memory CD4 T cells, monocytes, resting dendritic cells, and resting mast cells, which were related to a longer survival time. Correlated with worse prognosis, activated memory CD4 T cells and M0 macrophages were significantly enriched in the high-risk group.

Immune cell infiltration has been accepted to play an essential role in tumor progression and the response to immunotherapy in LUAD [44]. By eradicating tumor cells directly through cytolytic mechanisms or modulating the TME indirectly, CD4 + T cells can target tumor cells in various ways [45]. By helping to induce a gene expression program in CD8 + T cells that promotes cytotoxic T lymphocyte (CTL) function through various molecular mechanisms, CD4 + T cells assist CTLs in overcoming the barriers that sharply hinder antitumor immunity [46]. In addition, Probst, H C. et al. showed that peripheral CD8 + T-cell tolerance could result from antigen presentation by resting dendritic cells [47], revealing the vital role that resting dendritic cells play in immunotherapy resistance. On the other hand, numerous types of immune cells, comprising regulatory T cells, macrophages (M2), and terminally exhausted CD8 + T cells, have the potential to result in adverse clinical consequences due to their immune dysregulation [48]. These results indicated that patients in the high-risk group might have a better response to immunotherapy.

As a crucial part of immunotherapy, immune checkpoints can regulate T-cell effector function, bringing about breakthroughs and even constituting a paradigm shift in cancer therapy [49]. Recently, great efforts have been made to develop immune checkpoint blockade treatments, mainly targeting *PD-1, PD-L1*, and *CTLA-4* [50]. However, in contrast to LUSC patients, LUAD patients benefit little from *CTLA4* and anti-PD-1 or anti-PD-L1 therapy. Thus, improved ICI-based treatment approaches beyond those targeting the *CTLA4* and *PD-1/PD-L1* pathways are urgently needed. In this study, the mRNA expression levels of diverse immune checkpoints other than *PD-1, PD-L1*, and *CTLA-4* were analyzed with the TCGA database. The results showed that *CD27, IDO2, CD200R1, TNFRSF25, CD40LG, ADORA2A*, and *BTLA* were significantly enriched in the low-risk group and remarkably correlated with vital clinicopathologic features. Wang, Qinchuan et al. revealed that several immune points (including *BTLA, IDO,* and *CD27*) were optimal biomarkers for tumor recurrence and survival in renal cell carcinoma patients, and a high expression level of *BTLA* was also found to be related to decreased survival [51]. Acting as gatekeepers of the immune response, several inhibitory immunoreceptors have been identified and exploited in past decades, including *PD-1, CTLA-4, LAG3, TIM3, TIGIT* and *BTLA* [52]. As surface molecules, their activity can be easily restrained by blocking antibodies that inhibit ligand‒receptor engagement [53]. In addition, activating costimulatory T-cell receptors is deemed a promising therapeutic strategy in clinical practice [54]. Furthermore, the six immune checkpoints were identified as significantly connected with genes in our prognostic model as well as the risk score, implying that patients in the low-risk group are more suitable for immunotherapy based on costimulatory receptor targeting therapy.

Then, the tumor immune dysfunction and exclusion (TIDE) algorithm, which simulates two main immune escape mechanisms of tumors to predict the ICI response, was used to predict the response to immunotherapy [55]. The TIDE score has excellent performance in tumor immune escape prediction [56], which illustrates that patients with lower scores are more likely to share favorable responses to immunotherapy. In our study, the high-risk group had a lower TIDE score, representing more benefits from immunotherapy. However, as discussed before, patients in the high-risk group presented a lower immune score, suggesting that high immune cell proportions do not necessarily predict high immunogenicity.

Tumor mutation burden (TMB) has been deemed an efficient biomarker not only for measuring the number of mutations in a cancer but also for immunotherapy response [57, 58], where higher TMB tends to correlate with more promising outcomes from immunotherapy. To further elucidate the immune characteristics of LUAD patients, gene mutations were analyzed based on high- and low-risk subgroups, where missense mutations were most common. Various genomic alterations, including alterations in *EGFR, KRAS, ALK*, and *TP53*, have been proven to be related to ICI efficacy [13]. In particular, cooccurring mutations in *KRAS* and *TP53* have been determined to have predictive value in immune checkpoint inhibitors [59]. The top 20 genes with the highest mutation rates are displayed, among which TP53 shared the highest mutation frequency, with a higher level in the high-risk group. As reported before, TP53 was significantly correlated with oncogenic pathways, such as DNA replication, mismatch repair, and the cell cycle, contributing to undesirable clinical outcomes in LUAD patients [60]. In addition, mutations in *MET, KRAS*, and *TP53* have been revealed to sharply correlate with high *PD-L1* expression and a favorable ICI response [61]. These results corresponded with our observation that patients in the high-risk group had a worse prognosis but better immunotherapy responses. In addition, TMB was found to be positively associated with the risk score, further illustrating that patients in the high-risk group could benefit more from immunotherapy, and the prognostic model possesses excellent prediction value in measuring the TMB and immunotherapy response.

In contrast to bulk data that measure the averaged attributes of whole tissues, single-cell RNA sequencing (scRNA-seq) facilitates the identification of cell types and lineages of various cell subpopulations based on heterogeneous tissue ecosystems [62]. As discussed above, our prognostic model exhibited great efficacy in TMB measurement as well as prognosis and immunotherapy prediction. To explore the expression level of prognostic genes in different cell subpopulations, scRNA-seq was performed. The results showed that only BIRC5 was significantly enriched in T cells, which play a vital role in antitumor immunogenicity. Wang Y et al. revealed that baculoviral inhibitor of apoptosis protein (IAP) repeat containing 5 (BIRC5) expression can be regulated by the circCAMSAP1/miR-1182/BIRC5 axis, promoting NSCLC progression [63]. Besides, it has been revealed that the attenuation of the long non-coding RNA LINC00857 significantly augments the susceptibility of lung adenocarcinoma cells to radiotherapy, contingent upon BIRC5 expression, by inducing the recruitment of NF-κB1 [64]. As a well-known cancer therapeutic target, BIRC5 has been extensively researched, providing new insight into immunotherapy [65]. Based on these findings, we infer that BIRC5 could function as a biomarker and even therapeutic target in LUAD. Finally, the functional phenotype of BIRC5 was further explored by preliminary experiments. The significant BIRC5 mRNA levels in LUAD tissues and cell lines were confirmed by cell experiments, which was consistent with several findings obtained previously [66–68]. In addition, BIRC5 gene knockdown in LUAD cell lines was proven to significantly inhibit the activity and proliferation of cancer cells. The elevated expression of BIRC5 has been notably demonstrated to significantly facilitate tumorigenesis and migration, exerting a profound influence on the early detection and accurate prediction of the immunotherapeutic response in patients with LUAD.

In summary, an immune-related prognostic model was constructed based on the TCGA database to predict the OS of LUAD patients, which was validated by the GEO database. The risk score and clinical stage were found to be independent prognostic factors. The immunotherapy response was further analyzed, reflecting our model’s robust and capacious perspective in utilization. Unavoidably, deficiencies remain in our study because this is still a retrospective analysis. Thus, a prospective study or clinical samples and methods of animal models in vivo are needed to further confirm our results.

## Conclusions

We performed comprehensive bioinformatics analysis and identified a predictive model for LUAD prognosis based on thirteen immune-related genes. We assessed the prognosis and immunological microenvironment of LUAD patients via this model. By scRNA analysis, the expression of BIRC5 was identified significantly high in T cell. We also verified the role of BIRC5 in LUAD by cell assay, which may provide novel insight into LUAD patients management.

### Supplementary Information

**Additional file 1.**

**Additional file 2.**

**Additional file 3.**

**Additional file 4.**

## Data Availability

The data that support the findings of this study are available from the corresponding author upon reasonable request. We have uploaded all the raw data, code and images to the jianguoyun. This data is easily access at the following link: https://www.jianguoyun.com/p/DepODg4QjdemCxiAt_8EIAA

## Abbreviations

LUAD: Lung adenocarcinoma
IRmRNAs: Immune-related mRNAs
scRNA: Single-cell RNA sequencing
ALK: Anaplastic lymphoma kinase
EGFR: Epidermal growth factor receptor
ICIs: Immune checkpoint inhibitors
PD-1: Programmed cell death 1
PD-L1: Programmed cell death-ligand 1
CTLA-4: Cytotoxic T-lymphocyte-associated antigen 4
TME: Tumor microenvironment
TMB: Tumor mutation burden
TIDE: Tumor immune dysfunction and exclusion
IRGs: Immune-related genes
PCA: Principal component analysis
UMAP: Uniform Manifold Approximation and Projection
KEGG: Kyoto Encyclopedia of Genes and Genomes
GO: Gene Ontology
BP: Biological process
CC: Cellular component
MF: Molecular function
