# Supplementary material for: Establishing a prognostic model based on immune-related genes and identification of BIRC5 as a potential biomarker for lung adenocarcinoma patients
Source: BMC Cancer. 2023 Sep 23;23:897. doi: 10.1186/s12885-023-11249-8 (PMC10517491; doi:10.1186/s12885-023-11249-8)
Supplement: Supplementary file 1 — Additional file 1. [file 12885_2023_11249_MOESM1_ESM.docx]

**Supplementary Material**

**Supplementary Table S1** The clinical characteristics of both the training cohort and the test cohorts.

| **Variables** | **Group** | **Training set(n=468)** | **Testing set1 (n = 398)** | **Testing set2 (n = 114)** |
| --- | --- | --- | --- | --- |
| **Age** | **<=65** | 224 | 118 | 59 |
|  | **>65** | 244 | 280 | 55 |
| **Gender** | **Female** | 254 | 222 | 62 |
|  | **Male** | 214 | 176 | 52 |
| **Vital status** | **Alive** | 291 | 285 | 49 |
|  | **Dead** | 177 | 113 | 65 |
| **Survival time** |  | 911.709 | 791.907 | 1214.525 |
| **Clinical Stage** | **I** | 253 | 254 | 61 |
|  | **II** | 107 | 67 | 19 |
|  | **III** | 75 | 57 | 19 |
|  | **IV** | 25 | 15 | 2 |
|  | **unknow** | 8 | 5 | 13 |
| **T stage** | **T1** | 159 |  |  |
|  | **T2** | 248 |  |  |
|  | **T3** | 39 |  |  |
|  | **4** | 19 |  |  |
|  | **Tx** | 3 |  |  |
| **N stage** | **N0** | 302 |  |  |
|  | **N1** | 86 |  |  |
|  | **N2** | 66 |  |  |
|  | **N3** | 2 |  |  |
|  | **Nx** | 12 |  |  |
| **M stage** | **M0** | 315 |  |  |
|  | **M1** | 24 |  |  |
|  | **Mx** | 129 |  |  |

**Supplementary Table S2** The risk coefficient of the genes composing the risk signature

| **ID** | **coef** |
| --- | --- |
| IL7R | -0.354574449944973 |
| PTX3 | 0.280091939501254 |
| BIRC5 | -0.373376248816095 |
| PDGFB | 0.17844542061093 |
| ANGPTL4 | 0.133468185955199 |
| LIFR | -0.258775644800813 |
| SHC3 | -0.215478252276636 |
| TRIM6 | 0.19328619915441 |
| WNT3A | -0.344760553897506 |
| SCARF1 | -0.348614433237483 |
| FLI1 | 0.471347509779447 |
| PLK1 | 0.420796445345979 |
| C6 | -0.114590644226901 |
