# Supplementary material for: Establishing a prognostic model based on immune-related genes and identification of BIRC5 as a potential biomarker for lung adenocarcinoma patients
Source: BMC Cancer. 2023 Sep 23;23:897. doi: 10.1186/s12885-023-11249-8 (PMC10517491; doi:10.1186/s12885-023-11249-8)
Supplement: Supplementary file 2 — Additional file 2. [file 12885_2023_11249_MOESM2_ESM.docx]

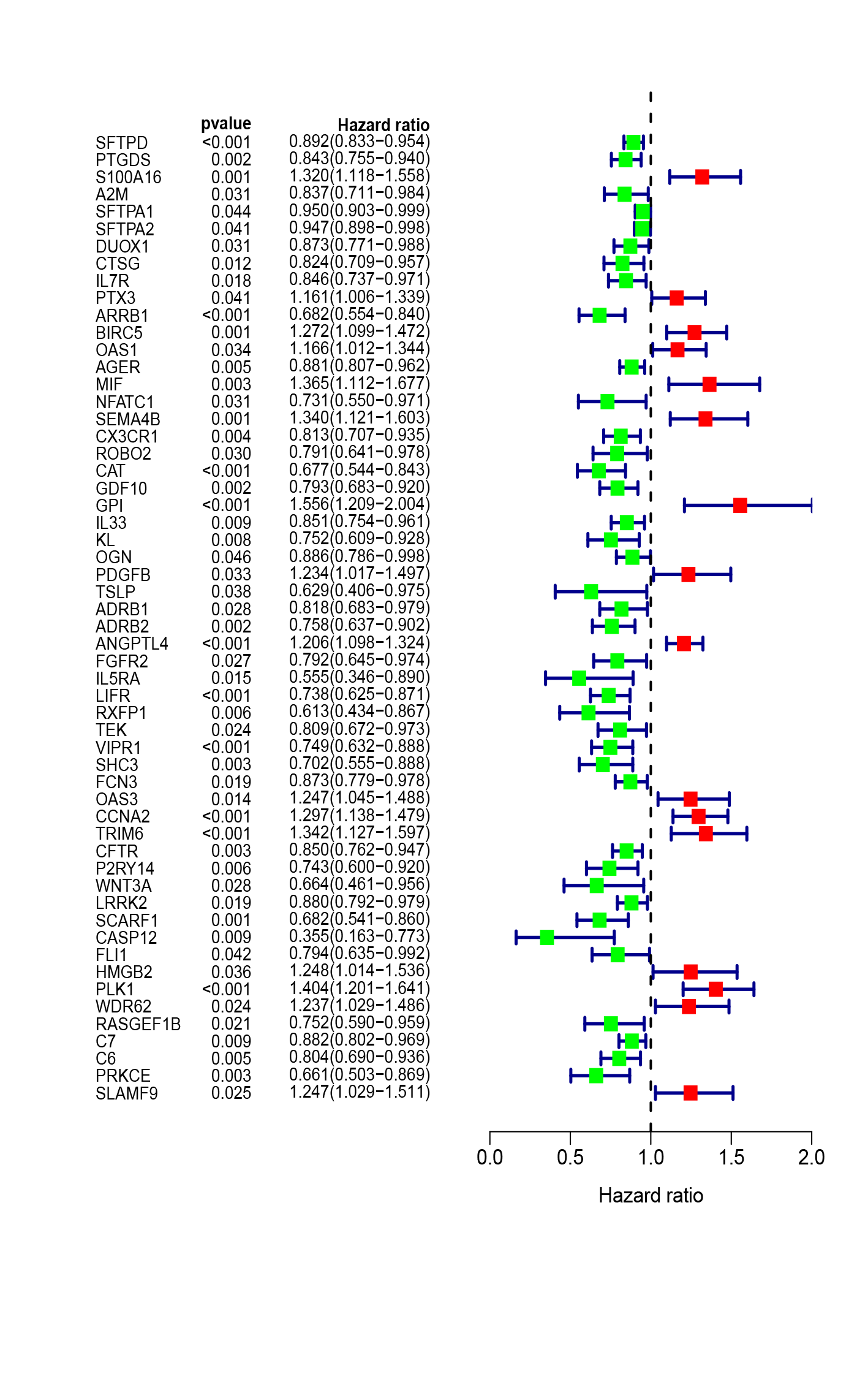
\

Supplementary Figure S1

Supplementary Material

**Supplementary Figure S1.** Results of the univariate Cox regression analysis.


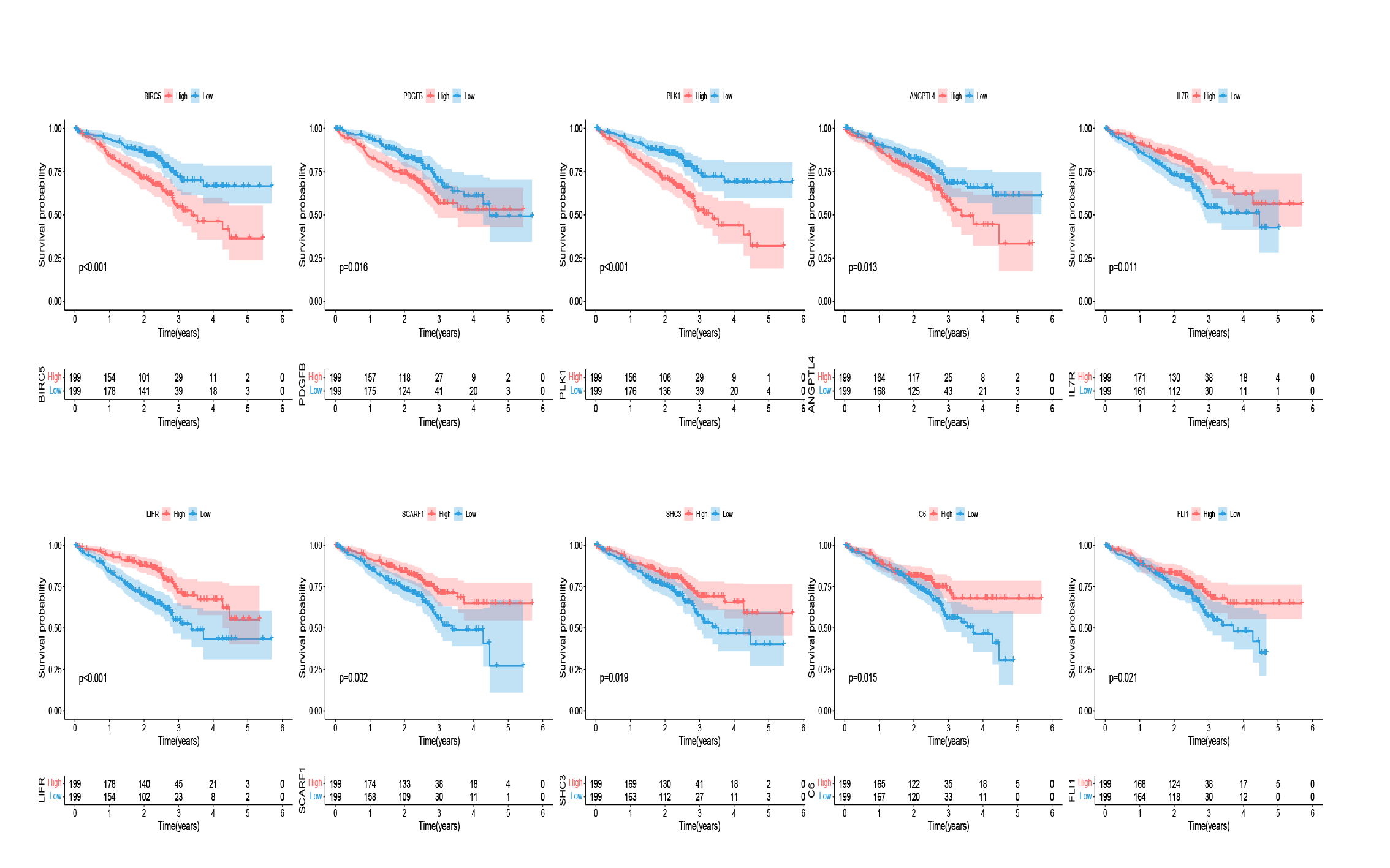


Supplementary Figure S2

**Supplementary Figure S2.** KM survival analysis of the prognostic genes from GSE72094.


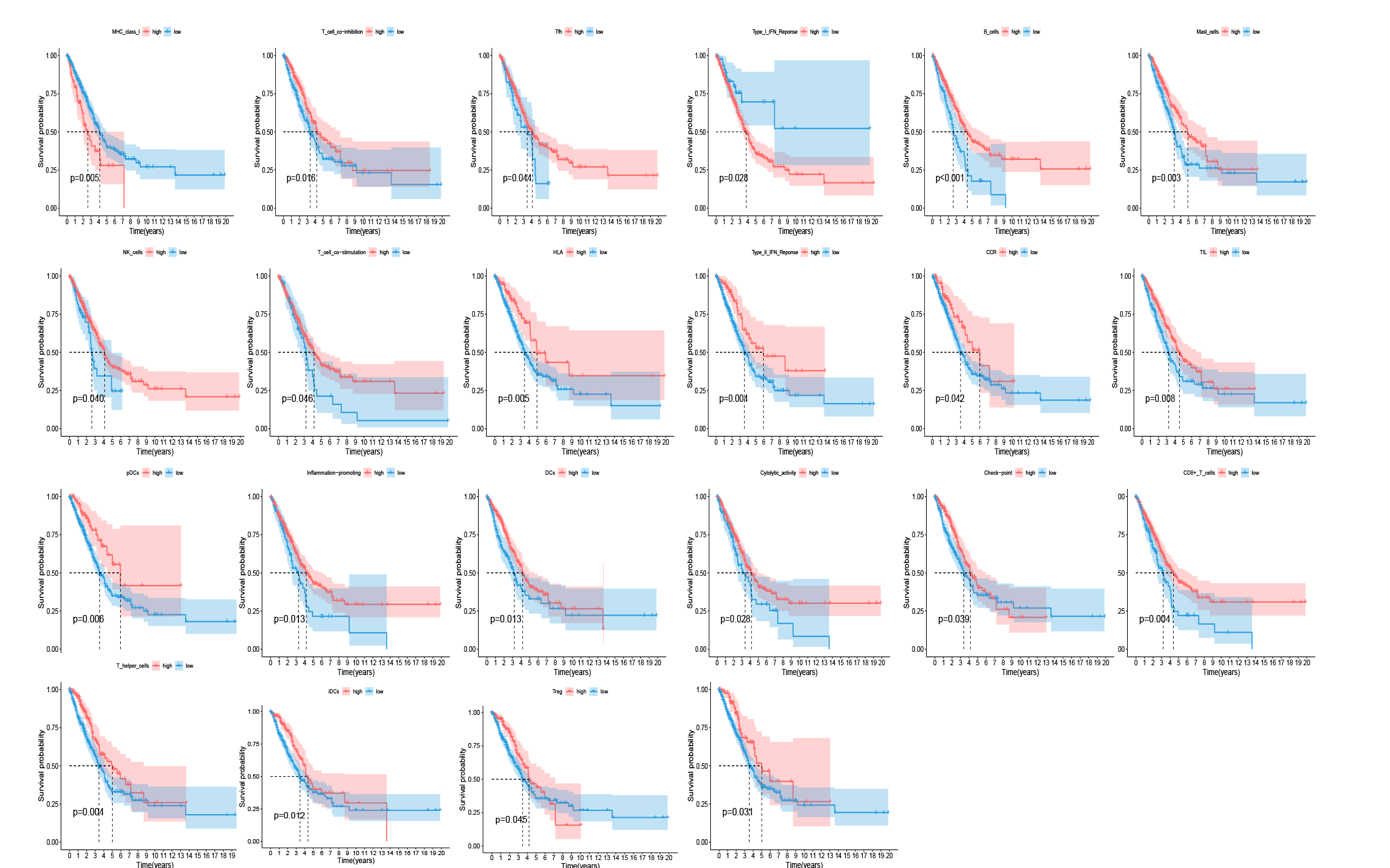


Supplementary Figure S3

**Supplementary Figure S3.** KM survival analysis of the immune-related functions. Consistent with the training cohort, BIRC5, PDGFB, PLK1, and ANGPTL4 were identified as the risk genes, while IL7R, LIFR, SCARF1, SHC3, C6, and FLI1 were confirmed as protective ones.

Supplementary Figure S4


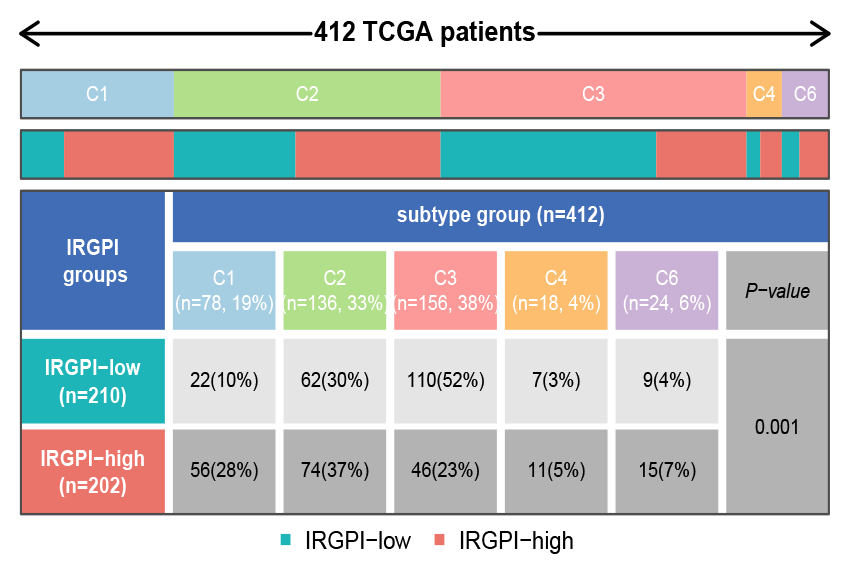


**Supplementary Figure S4.** [Immunophenotyping](javascript:;) based on patients from high-and-low risk groups.
