# Supplementary figures and images for: Establishing a prognostic model based on immune-related genes and identification of BIRC5 as a potential biomarker for lung adenocarcinoma patients
Source: BMC Cancer. 2023 Sep 23;23:897. doi: 10.1186/s12885-023-11249-8 (PMC10517491; doi:10.1186/s12885-023-11249-8)

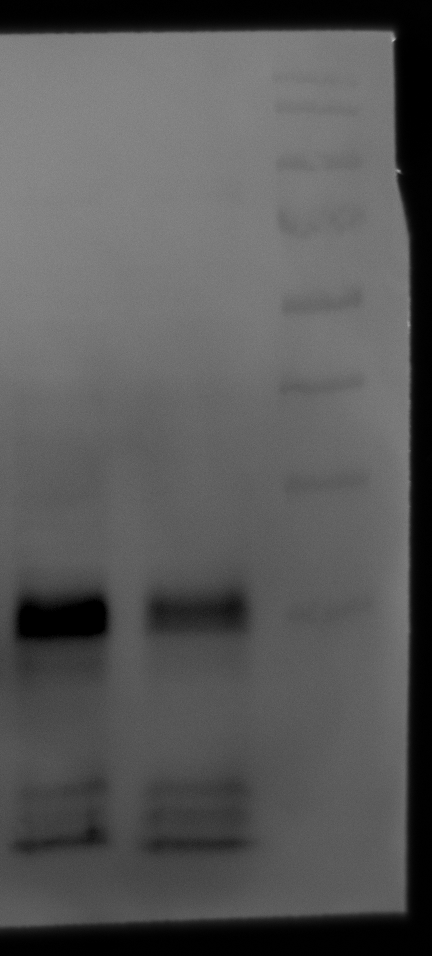

Supplement: Supplementary file 3 — Additional file 3. [file 12885_2023_11249_MOESM3_ESM.tif]

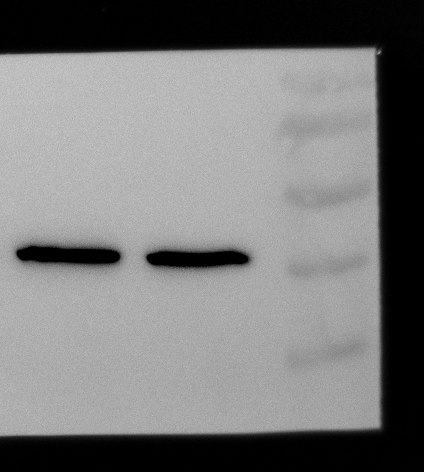

Supplement: Supplementary file 4 — Additional file 4. [file 12885_2023_11249_MOESM4_ESM.tif]
